# *De novo* sequencing and comparative transcriptome analysis of the male and hermaphroditic flowers provide insights into the regulation of flower formation in andromonoecious *taihangia rupestris*

**DOI:** 10.1186/s12870-017-0990-x

**Published:** 2017-02-28

**Authors:** Weiguo Li, Lihui Zhang, Zhan Ding, Guodong Wang, Yandi Zhang, Hongmei Gong, Tianjun Chang, Yanwen Zhang

**Affiliations:** 1 College of Life Science, Changchun Normal University, Changchun, 130032 Jilin China; 2 College of Resource and Environment, Henan Polytechnic University, Jiaozuo, 454000 Henan China

**Keywords:** Male flower, Hermaphroditic flower, Flower formation, Sex differentiation, *Taihangia*, Transcriptome

## Abstract

**Background:**

*Taihangia rupestris*, an andromonoecious plant species, bears both male and hermaphroditic flowers within the same individual. However, the establishment and development of male and hermaphroditic flowers in andromonoecious *Taihangia* remain poorly understood, due to the limited genetic and sequence information. To investigate the potential molecular mechanism in the regulation of *Taihangia* flower formation, we used *de novo* RNA sequencing to compare the transcriptome profiles of male and hermaphroditic flowers at early and late developmental stages.

**Results:**

Four cDNA libraries, including male floral bud, hermaphroditic floral bud, male flower, and hermaphroditic flower, were constructed and sequenced by using the Illumina RNA-Seq method. Totally, 84,596,426 qualified Illumina reads were obtained and then assembled into 59,064 unigenes, of which 24,753 unigenes were annotated in the NCBI non-redundant protein database. In addition, 12,214, 7,153, and 8,115 unigenes were assigned into 53 Gene Ontology (GO) functional groups, 25 Clusters of Orthologous Group (COG) categories, and 126 Kyoto Encyclopedia of Genes and Genomes (KEGG) pathways, respectively. By pairwise comparison of unigene abundance between the samples, we identified 1,668 differential expressed genes (DEGs), including 176 transcription factors (TFs) between the male and hermaphroditic flowers. At the early developmental stage, we found 263 up-regulated genes and 436 down-regulated genes expressed in hermaphroditic floral buds, while 844 up-regulated genes and 314 down-regulated genes were detected in hermaphroditic flowers at the late developmental stage. GO and KEGG enrichment analyses showed that a large number of DEGs were associated with a wide range of functions, including cell cycle, epigenetic processes, flower development, and biosynthesis of unsaturated fatty acid pathway. Finally, real-time quantitative PCR was conducted to validate the DEGs identified in the present study.

**Conclusion:**

In this study, transcriptome data of this rare andromonoecious *Taihangia* were reported for the first time. Comparative transcriptome analysis revealed the significant differences in gene expression profiles between male and hermaphroditic flowers at early and late developmental stages. The transcriptome data of *Taihangia* would be helpful to improve the understanding of the underlying molecular mechanisms in regulation of flower formation and unisexual flower establishment in andromonoecious plants.

**Electronic supplementary material:**

The online version of this article (doi:10.1186/s12870-017-0990-x) contains supplementary material, which is available to authorized users.

## Background

Flowering plants display remarkably variable floral architectures, which largely contribute to the diversity of sexual systems in angiosperms [1]. Andromonoecy is a rare sexual system, in which an individual bears both staminate and perfect flowers. In plant kingdom, it has been found in nearly 4000 plant species, comprising 1.7% of flowering plants [2]. As a transitional stage from hermaphroditism to monoecy, andromonoecy has independently evolved many times in different plant groups [3]. Given the important position in the evolution of plant sexual system, the developmental and genetic mechanisms underlying unisexual flower determination have attracted considerable attention in recent years [4–6].

*Taihangia rupestris* Yu & Li (Rosaceae), an endangered perennial herb belonging to the tribe Dryadeae, is only disjunctively and sporadically distributed on cliff faces in the southern part of the Taihang Mountains of China [7]. Unlike other genera of this tribe, such as *Dryas* and *Geum*, *Taihangia* always produces both bisexual flowers and unisexual male flowers in the same individual (Additional file 1: Figure S1), forming the andromonoecious sexual system [8]. *Taihangia* unisexual male flowers are bisexual at initiation and become unisexual by arresting the pistil development in consequent developmental stages. During the initiation of floral primordia, temperature is crucial for establishment of the uni- or bisexual flowers. Under low temperature conditions, the pistils of the bisexual flowers tend to abort, resulting in a high proportion of unisexual male flowers [9]. Several MADS-box genes involved in the floral organ development in *Taihangia*, including B-, C-, D-, and E-Class genes, have been identified in previous studies [10–12]. Of these identified MADS genes, class B MADS-box gene *TrPI* was expressed in petals and stamens, while class E gene *TrSEP3* was strongly expressed in the three inner whorls [10, 11]. For Class C gene *TrAG*, it was initially expressed in the floral meristem domain that will initiate stamens and carpels, when the stamen primordia are firstly observed. At the late stage of carpel development, *TrAG* is mainly expressed in the ovules, developing styles, and stigmas [12]. Despite the progresses mentioned above, underlying molecular mechanisms in the floral organ development and regulation of flower formation in *Taihangia* remain poorly understood, due to limited genetic and sequence information for this non-model plant species.

Currently, the next generation sequencing (NGS) technology provides opportunities for the efficient and comprehensive analysis of gene expression at whole-genome level in non-model plant without a reference genome [13]. The powerful NGS technology has been widely applied to comparative transcriptome studies of male and female flowers on large-scale gene expression profiles in some plant species, generating considerable genomic data for the identification of functional genes involved in sex differentiation and flower development [14–16]. However, the knowledge about unisexual male flower specification and development in andromonoecious plant is still limited, to some extent, due to its infrequent occurrence. Up to now, only a few transcriptome studies have been performed for andromonocious plant species [6, 17, 18].

To investigate the possible molecular mechanisms in the regulation of flower formation within andromonoecious *Taihangia*, we performed *de novo* transcriptome sequencing of the male and hermaphroditic flowers at both early and late developmental stages by using the Illumina platform. Based on the differences in unigene abundance among sequencing libraries, we identified the differentially expressed genes (DEGs) between the male and hermaphroditic flowers at early and late developmental stages, respectively. Furthermore, GO and KEGG enrichment analyses were conducted to elucidate the main functions of DEGs, and to identify candidate genes involved in the regulation of flower formation for further functional analysis. The present study would provide a genomic resource for gene discovery in the future and shed light on the underlying molecular mechanisms responsible for establishment of unisexual flowers and sex differentiation in andromonocious plant *Taihangia*.

## Methods

### Plant material and RNA extraction

**Fig. 1:**
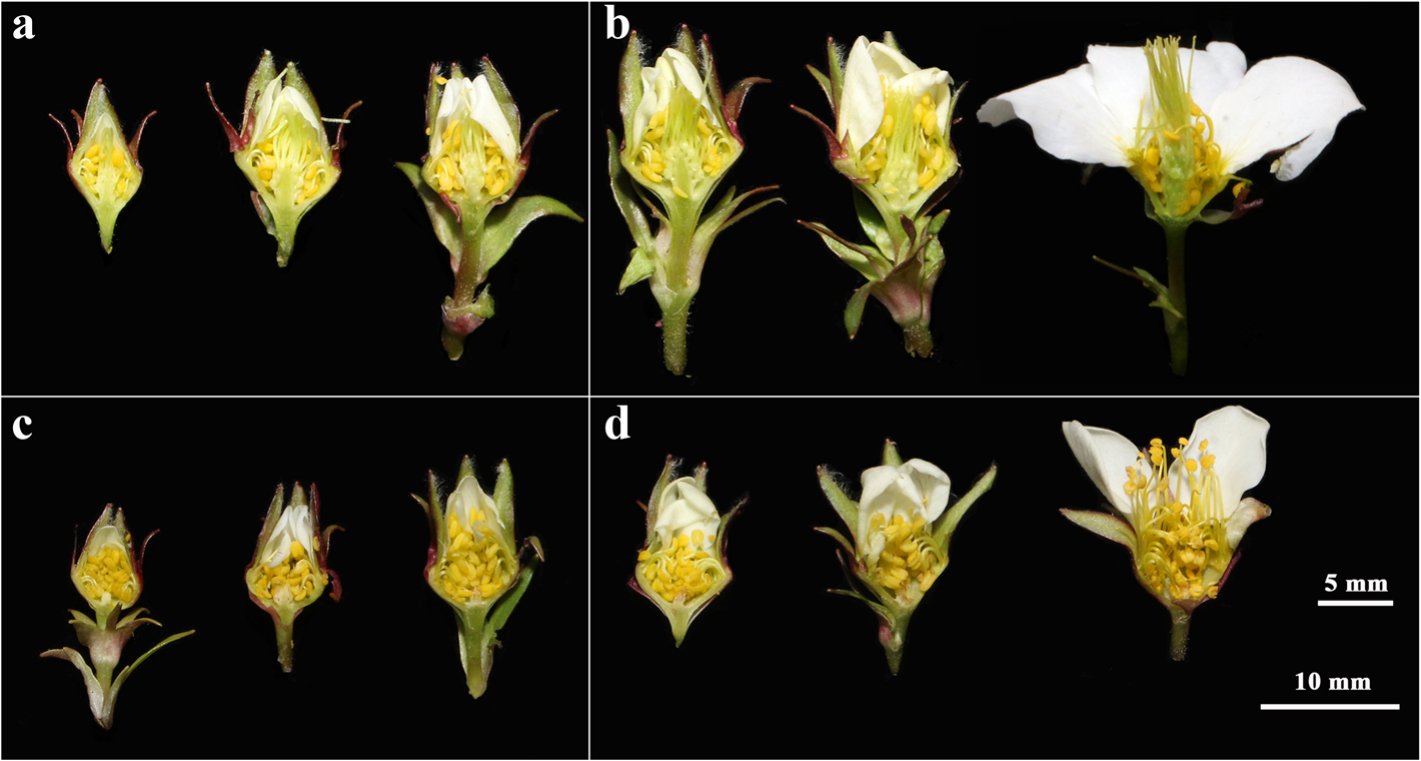
Male and hermaphroditic flowers within andromonoecious *Taihangia* at early and late developmental stages used in RNA-seq. **a** Hermaphroditic floral bud (EH); (**b**). Hermaphroditic flower (LH); (**c**) Male floral bud (EM); (**d**) Male flower (LM). Early developmental stage: young and elongated buds; late developmental stage: young flowers at pre-anthesis, flowers at anthesis, and fully opened flowers

The plant materials were transplanted from Yidoushui (35°28′ N, 113°23′E) and Zhuyufeng (35°27′ N, 113°22′E), Henan, China in April 2014, and were grown in the greenhouse of Henan Polytechnic University. In early spring 2015, the early and late developmental stages of male and hermaphroditic flowers, including male floral bud (EM), hermaphroditic floral bud (EH), male flower (LM), and hermaphroditic flower (LH), were harvested (Fig. 1). As shown in Fig. 1, flowers were classified into six developmental stages: young flower buds (stage A, < 0.5 cm), elongated buds (stage B, 0.5-1 cm and stage C, >1 cm), pre-anthesis (stage D), anthesis (stage E, flowers with partially opened petals), and fully opened flowers (stage F). In this study, the flower samples at early developmental referred to flower buds including young and elongated buds (stage A-C). The flower samples at late developmental stage were comprised of young flowers at pre-anthesis (stage D), flowers at anthesis (stage E), and mature flowers with fully opened petals (stage F). The samples of the male and hermaphroditic flowers were collected from six different individuals at each of the developmental stage (stage A-F), respectively, and immediately frozen in liquid nitrogen and stored at −80 °C for RNA extraction.

Total RNA was isolated from 100 mg homogenized plant material using an RNeasy Mini Kit (Qiagen, Germany) for plant tissues according to the manufacturer’s manuals. RNA quality was preliminarily assessed by 1.5% agarose gel electrophoresis, and then quantified with a UV-visible spectrophotometer (UV-2550, Japan). RNA samples for further analysis were selected according to the following criteria: A260/A280 ratio was 1.9-2.1 and A260/A230 ratio ranged from 2.0 to 2.5. The equal amounts of RNA from male flower buds at each of the early developmental stages (stage A-C) were mixed as one EM sample, while one LM sample was prepared by combinations of the identical amounts of RNA from male flowers at each of the late developmental stages (stage D-F). Similarly, EH and LH samples were obtained using the same method described above. In total, 12 RNA samples with three biological replicates were prepared for cDNA synthesis and qPCR analysis. After that, specific RNA pools of EM, EH, LM, and LH were obtained by combining identical quantities of total RNA from the corresponding biological triplicates. Quality and quantity of the pooled RNA were further analyzed using a Nanodrop 2000 instrument (Thermo Scientific) and a Chip RNA 7500 Series II Bioanalyzer (Agilent), respectively. RNA samples with 260/280 ratios (range 1.9 to 2.1), 260/230 (range 2.0 to 2.5) and RIN (RNA integrity number) more than 8.0 were used to cDNA library construction and Illumina sequencing.

### Library construction and RNA-Seq

Construction of the four libraries and the RNA-Seq were performed by the Biomarker Biotechnology Corporation (Beijing, China). Five micrograms of total RNA from each sample (EM, EH, LM, and LH) were used to construct the sequencing libraries. RNA sequencing libraries were generated using the TruSeq RNA Sample Prep Kit (Illumina, San Diego, CA) following the manufacturer’s recommendations. Briefly, the poly (A)-containing mRNA molecules were purified from the total RNA by using the poly-T oligo-attached magnetic beads (Illumina, San Diego, CA, USA). Then, mRNA was broken into short fragments, which were used as templates for cDNA synthesis. Double-stranded cDNA was synthesized using SuperScript II, buffer, dNTPs, RNaseH, DNA polymerase I, and random hexamer primers (Illumina). After that, the ‘A’ tail was added to the 3′ ends of the repaired cDNA fragments and the Illumina’s paired-end adapters were ligated to the cDNA ends. The products from the ligation reaction were amplified by PCR. The PCR productions were purified by magnetic beads (Illumina) and dissolved in EB solution to generate the sequencing libraries. The quantity and quality of each sequencing library were tested by the Agilent 2100 Bioanalyzer. Finally, the four libraries were sequenced separately on Illumina HiSeq™ 2500 platform.

### Sequence assembly and gene annotation

After sequencing, paired-end raw reads were firstly processed through in-house perl scripts [19, 20]. In this step, clean reads were acquired by removing the reads with adaptor contamination, low-quality sequences (reads with ambiguous ‘N’ bases larger than 5%), and reads with more than 10% Q < 20 bases. At the same time, Q20, Q30, and GC-content of the clean data were calculated. All the downstream analyses were based on clean data with high quality. *De novo* assembly of the transcriptome was performed by Trinity software (release 2014-07-17) [13], with default parameters and no reference sequence. In brief, reads were assembled into the contigs by the Inchworm program at the first step. The minimally overlapping contigs were clustered into connected components by the Chrysalis program, and then the transcripts were constructed by the Butterfly program [14]. Finally, the transcripts were further clustered based on nucleotide sequence identity, and the longest transcripts in the cluster units were regarded as unigenes to eliminate redundant sequences. For detection of transcriptional profiles in the male and hermaphroditic flowers at different stages, clean reads from each library were initially assembled separately. To obtain a uniform transcriptome reference across samples, all clean reads from four libraries were pooled together and *de novo* assembled to generate unigenes for assembly evaluation, gene annotation, and expression analysis. The raw sequencing data were deposited in the NCBI Short Read Archive (SRA) database (http://www.ncbi.nlm.nih.gov/sra/) under the accession number SRP081195.

To identify putative functions of *Taihangia* unigenes, we performed functional annotation by using a BLASTx search (E-value ≤ 10^−5^) against a series of protein databases: the National Center for Biotechnology Information non-redundant (Nr), SwissProt, Pfam, Clusters of Orthologous Groups (COG), Gene Ontology (GO), and Kyoto Encyclopedia of Genes and Genomes (KEGG) databases. The Blast2GO software package was used to compare and determine the unigene GO annotations [21]. Finally, WEGO software was used to obtain the GO functional classifications for all annotated unigenes [22].

### Transcription factors (TFs) prediction

In order to identify the TFs represented in *Taihangia* transcriptome, all assembled unigenes were searched against the plant transcription factor database PlantTFDB 4.0 by using blastX with a cut-off E-value of 1 e^−5^ [23]. To facilitate the transcription factor prediction, the identified TFs were further screened with similarity ≥ 0.6 and coverage ≥ 0.4.

### Analysis of differentially expressed genes (DEGs)

To calculate the amount of gene expression for each unigene, RSEM [24] was used to quantify the number of reads mapped to the assembled transcriptome, and read count for each gene was obtained from the mapping results. We used the FPKM [25] (fragments per kilobase of gene per million mapped reads) algorithm to normalize the gene expressional abundances in each library. By pairwise comparisons of the four libraries, the DEGs were identified using the DEGseq (1.18.0) R package based on the read counts for each gene at different libraries [26]. In addition, the false discovery rate (FDR) control method [27] was used to identify the threshold of the *P*-value in the significance tests. Significance of differential expressed genes was defined at a false discovery rate < 0.01 and an absolute value of log_2_-ratio ≥ 2. The classification of DEGs were performed with GO and KEGG analysis using the method described above. GO enrichment analysis of the differentially expressed genes (DEGs) was implemented by using the Cytoscape plugin BiNGO using a hypergeometric test with a corrected *P*-value < 0.05 [28]. KEGG pathway enrichment analysis of the DEGs was performed by using KOBAS with the hyper-geometric distribution model [29]. The enrichment *p*-values were adjusted by using the Benjamin and Hochberg method.

### Validation of sequencing data by quantitative real-time PCR (qPCRs)

Sixteen DEGs were randomly selected and the expression profiles were investigated by qRT-PCR to confirm the transcriptome data. First-strand cDNA was synthesized from the same RNA samples used for library construction by using the PrimeScript RT reagent Kit with gDNA Eraser (Perfect Real Time) (Takara, Japan) according to the instructions. Quantitative real-time PCR (qRT-PCR) was performed by using the SsoFast EvaGreen Supermix RT-PCR kit (Bio-Rad Laboratories) and the MiniOpticon Real-Time Detection System (Bio-Rad). The PCR mix was composed of 10 μL EvaGreen Supermix, 2.0 μL of 1:10 diluted cDNA, 0.5 μL of each primer (10 mM), and 7.0 μl water in a final volume of 20 μL. The reactions were incubated under following cycling conditions: 2 min at 50 °C, 2 min at 95 °C, 40 cycles of 95 °C for 15 s, 58 °C for 15 s, and 72 °C for 30s, and finally 72 °C for 2 min. After the final cycle, a melting curve analysis was performed with a single cycle from 60 °C to 95 °C in 5 s intervals to verify the reaction specificity. The efficiency of the primer sets was calculated by performing real-time PCR on serial dilutions of first-strand cDNAs templates [30]. By using the geNorm software [31], EF1α and UBQ were identified as reference genes for stable expression across developmental stages. The relative expression of each gene was calculated using the standard E^-ΔΔCq^ method [32], and normalized with the internal standard gene EF1α and UBQ. Expression quantification and data analysis were performed according to MIQE guideline suggested by Bustin et al. [33]. Three independent biological replicates and three technical repetitions were performed for each of the quantitative PCR experiments. The relative expression of each target gene was analyzed by one-way ANOVA. Statistical analyses of the data were performed at *P* < 0.05 significance level using SPSS 16.0 software.

## Results

### Illumina sequencing and *de novo* assembly

To obtain a comprehensive flower transcriptome in andromonoecious *Taihangia*, four cDNA libraries (EM, EH, LM, and LH) were constructed from male and hermaphroditic flowers covering early and late development stages. Paired-end sequencing of the four constructed libraries were performed on an Illumina Hiseq 2500 platform, generating a total of 90,806,187 raw reads. After filtering for low quality region, adapters, and sequencing tags, we obtained 84,596,426 qualified Illumina reads (93.16%), including 21,136,736 (93.36%), 20,963,888 (92.81%), 21,096,120 (93.16%), and 21,399,682 (93.32%) clean reads for the libraries of EM, EH, LM, and LH, respectively (Table 1). Overall, the library produced 21.31G base pairs clean data with 91.43% Q30 bases (percentage of sequences with sequencing error rates < 0.1%), indicating that the throughput sequencing was accurate enough to warrant the further analysis. The clean reads from each libraries were separately *de novo* assembled by Trinity programs, generating 42,630 (EM), 41,419 (EH), 38,764 (LM), and 39,857 (LH) unigenes, respectively, with mean lengths of 855 to 905 bp. After that, we pooled all high quality reads from the four individual libraries to perform the *de novo* assembly for a uniform reference transcriptome. By using the Trinity program, a total of 59,064 unigenes longer than 200 bp were assembled with an N50 length of 1,594 bp, an average length of 840 bp, and a maximal transcript length of 11,665 bp. The length distribution of *Taihangia* unigenes was shown in Additional file 2: Figure S2.

**Table 1:** Summary of Illumina transcriptome sequencing and assembly for *Taihangia rupestris* flower

	EM	EH	LM	LH	EM + EH + LM + LH
Number of raw reads	22,638,962	22,588,764	22,645,959	22,932,502	
Number of clean reads	21,136,736 (93.36%)	20,963,888 (92.81%)	21,096,120 (93.16%)	21,399,682 (93.32%)	84,596,426
Number of base pairs	5,325,126,434	5,281,720,198	5,314,989,939	5,391,407,889	21,313,244,460
GC (%)	46.42	46.74	46.29	46.54	46.50
Q20 (%)	97.41	97.34	97.39	97.44	97.40
Q30 (%)	91.47	91.27	91.42	91.59	91.43
Number of contigs	2,247,974	2,222,351	2,178,823	2,292,464	8,230,709
Number of transcripts	117,759	115,373	102,649	110,462	167,441
Total unigenes generated	42,630	41,419	38,764	39,857	59,064
N_50_ length (bp)	1,541	1,561	1,570	1,611	1,594
Average unigene length (bp)	855	878	886	905	840

To evaluate the quality of *de novo* assembly without a reference genome, a common method is to calculate the percentage of reads that can be mapped back to unigene sets. Based on this metric, we found that more than 84% of clean reads were mapped back to the *Taihangia* unigenes for each library (Additional file 3: Table S1). In addition to in silico quality assessment, the standard PCR and Sanger sequencing were conducted to evaluate the quality and accuracy of *Taihangia* assembled unigenes. Of 18 designed primer pairs, 17 primer pairs for 16 unigenes could steadily generated expected PCR products. All 17 PCR products were sequenced by Sanger sequencer, and the obtained sequences were compared with the assembled unigenes. The alignment analysis showed that the sequences from Sanger sequencing met well with the assembled unigenes with an average sequence identity of 98.7%, confirming the accuracy of assembled unigenes derived from transcriptome (Additional file 4: Table S2). Together, our results supported the high quality of *de novo* assembly in current sequencing data.

### Functional annotation and classification of the unigenes

All the assembled 59,064 unigenes were subjected to BlastX comparisons, setting a cut-off E-value of 10^−5^, against the public databases including Nr, Pfam, Swiss-Prot, GO, KEGG and COG databases. As a result, a total of 25,231 unigene sequences showed significant similarity to known proteins in publicly available databases (Table 2). Based on the annotation results of the Nr database, the E-value distribution analysis showed that 63.02% of the matched sequences had strong homology with the E-value < 1 e^−50^, while 36.98% of the matched sequences ranged between 1e^−5^ and 1e^−50^. The similarity distribution analysis revealed that 58.29% of the unigenes had a similarity higher than 75%, while 22.36% of mapped sequences had a similarity ranging from 60% to 75%. For species distribution, the Nr database queries showed that 68.03% of the *T. rupestris* annotated sequences were matched to the sequences of *Fragaria vesca*, followed by *Prunus mume* (6.43%), *Prunus persica* (6.41%), *Malus domestica* (4.46%), and *Pyrus × bretschneideri* (3.30%). Characteristics of the homology search of *Taihangia* unigenes against the NR database were shown in Additional file 5: Figure S3.

**Table 2:** Summary for the annotation of unigenes of *Taihangia* flower

Annotated databases	Unigene	300 ≤ length < 1000 nt	≥1000 nt
KEGG	8,115	2,215	5,000
COG	7,153	1,358	5,293
GO	12,214	3,365	7,508
KOG	13,310	3,693	8,144
Pfam	17,055	4,214	11,526
Swissprot	15,978	4,459	9,838
Nr	24,753	7,946	13,520
All	25,231	8,106	13,557

Based on the BLASTx results against the Nr database, we added GO terms to obtain GO functional annotations and categorizations of these assembled unigenes. Of the 24,753 unigenes with Nr annotation, a total of 12,214 unigenes with at least one GO term were assigned into biological processes (24,571 unigenes), cellular components (10,195 unigenes), and molecular functions (18,239 unigenes) categories. The three main categories were further classified into 53 functional groups including 20 biological processes, 17 cellular components and 16 molecular functions. In the category of molecular function, “catalytic activity” was the most highly represented group, followed by group of “binding”. Under the biological process category, “metabolic process” and “cellular process” were the most highly represented groups. For the cellular component, the major groups were “cell” and “cell part”. The GO analysis indicated that a great number of identified unigenes were associated with various biological processes and molecular functions in *Taihangia* floral tissues. The annotated sequences were further subjected to a search against the COG database for functional prediction and classification. As a result, 7,153 unigenes were grouped into 25 COG classifications, among which the “General Function prediction only” represented the largest group (2,025 unigenes), followed by “Replication, recombination and repair” (1,186 unigenes), “Transcription” (1,060 unigenes), “Signal transduction mechanisms” (904 unigenes), and “Posttranslational modification, protein turnover, chaperones” (724 unigenes). The category “Nuclear structure” with 3 unigenes was the smallest group. To obtain a better understanding of biological functions of the unigenes, the annotated sequences were searched against the KEGG database. Among the 25,231 annotated unigenes, 8,115 had significant matches and were assigned to 126 KEGG pathways. The top five pathways were “Ribosome” (ko03010), “Protein processing in endoplasmic reticulum” (ko04141), “Carbon metabolism” (ko01200), “Biosynthesis of amino acids” (ko01230) and “Spliceosome” (ko03040). Functional GO, COG, and KEGG pathway annotations and classification of the unigenes were shown as Additional file 6. In summary, all these annotation and classification analyses would provide valuable information for gene discovery and functional genomic studies in the future.

### Identification of transcription factors

**Fig. 2:**
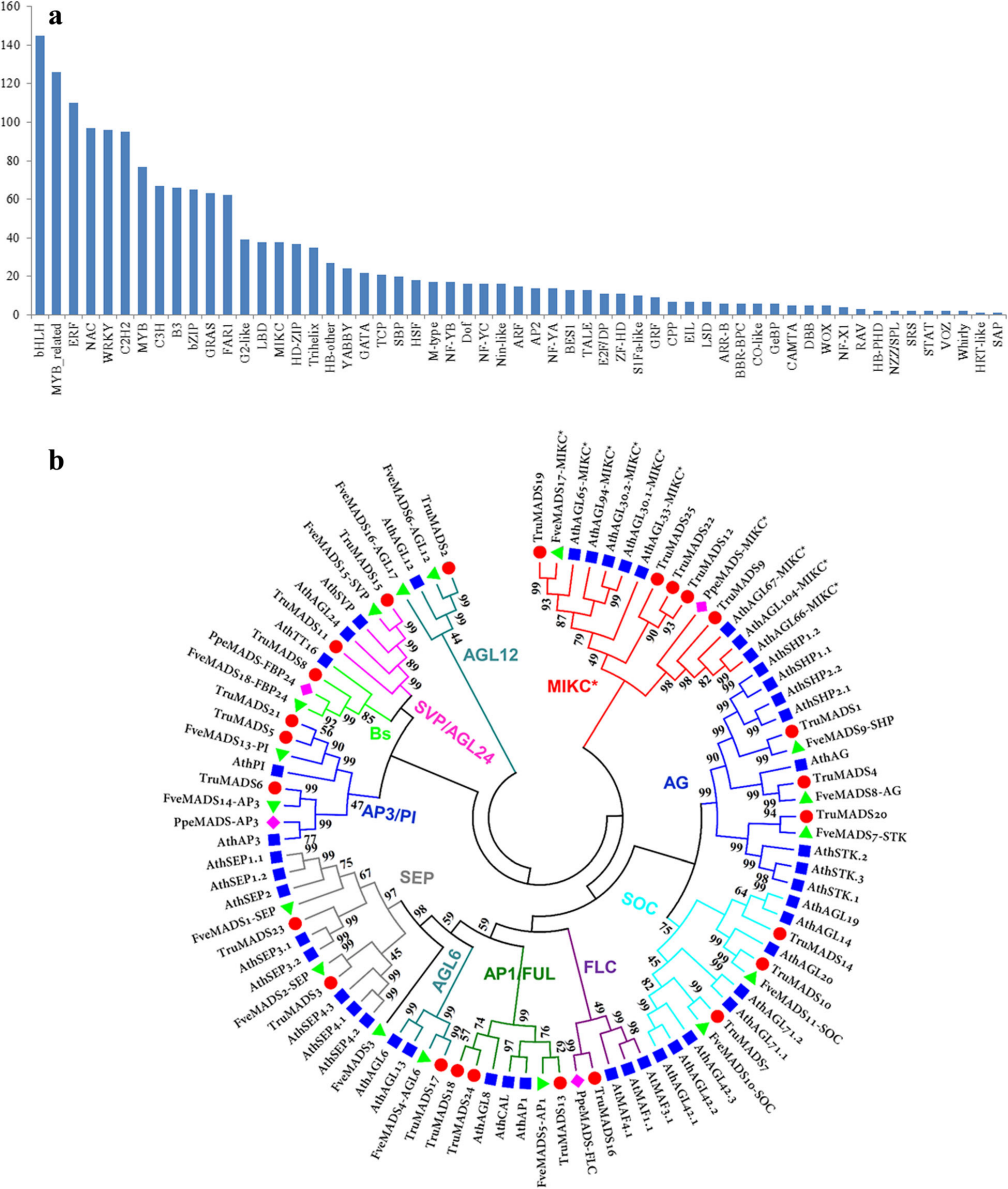
Identification of transcription factors (TF) and MADS-box genes in *Taihangia*. **a** Classification of TF families. **b** Phylogenetic tree of MIKC MADS-box proteins of *Taihangia*, *Arabidopsis thaliana*, strawberry, and peach. *Red dots*, *Taihangia* genes; *green dots*, strawberry genes; *blue dots*, *A. thaliana* genes; *purple dots*, peach genes

In the present study, we identified 1,661 TFs, representing 1.95% of *Taihangia* unigenes and falling into 57 TF families classified by plant transcription factor database PlantTFDB 4.0 (Fig. 2a, Additional file 7). Among these detected TF gene families, bHLH (145) was the most abundant TF family, followed by MYB_related (126), ERF (110), NAC (97), WRKY (96), and C2H2 (95). Notably, a total of 55 MADS-box genes, including 38 MIKC-type and 17 M-type TFs, were found in *Taihangia* based on transcriptome data.

To examine the phylogenetic relationships among the *Taihangia* MADS-box genes and to classify them into the established subfamilies, a Neighbor–Joining (NJ) phylogenetic tree was constructed based on a multiple sequence alignment of the predicted full-length MADS-box protein sequences of *F. vesca*, *P. persica*, and *Arabidopsis* (Fig. 2b). The phylogenetic analysis revealed that 25 MADS-box TFs, including eight MADS-box genes reported in previous studies, were classified into classified into 10 MIKC^c^ and one MIKC* subfamilies (Additional file 8: Table S3). Furthermore, several MADS-box TFs, such as APETALA3 (AP3), PISTILLATA (PI), AGAMOUS (AG), SHATERPROOF (SHP), and SEPALLATA (SEP), described in ABCDE model were also included. TruMADS13, TruMADS18, and TruMADS24 belonged to the AP1/FUL subfamily in the A class, which included the AP1and FUL clades. TruMADS13 was classified into AP1 clade, while TruMADS18 and TruMADS24 were sorted into FUL clade. In the functional B-class (AP3/PI) group, two genes (TruMADS5 and TruMADS21) belonged to PI clade, while the other one gene (TruMADS6) fell within TM6. Three MADS-box TFs, TruMADS1, TruMADS4, and TruMADS20, were assigned to the AG family, which were functionally classified as a C/D class group. TruMADS4 belonged to the C-class group AG while TruMADS1 and TruMADS20 were most orthologous to the D-class functional group SHP and STK, respectively. In the E functional group, two MADS-box TFs, TruMADS3 and TruMADS23, were assigned to SEP subfamily. In general, the phylogenetic analysis showed that *Taihangia* contains MADS proteins in each putative functional group.

### Transcriptome profiles in male and hermaphroditic flowers

Differences of gene expression profiles between male and hermaphroditic genders at different developmental stages were examined by pairwise comparisons of the four libraries (EM, EH, LM, and LH). A total of 36,520 unigenes (53.26%) were shared among all libraries, while approximate 15.51% of unigenes (10,639 unigenes) was detectable in individual libraries (Additional file 9: Figure S4). For hermaphroditic genders, there were 2,176 and 2,475 unigenes exclusively expressed in EH and LH, respectively. Meanwhile, we detected 3,125 unigenes specific for EM and 2,863 unigenes only for LM. A total of 12,829 unigenes (18.71%) were gender-specific, with 6,304 unigenes detected only in the male flowers (EM + LM) and 6,525 unigenes in hermaphroditic flowers (EH + LH), respectively. In addition, 14.06% of assembled unigenes (9,641 unigenes) was only detected in floral buds (EM + EH), whereas 9.77% of assembled unigenes (6,702 unigenes) was observed in the mature flowers (LM + LH), but not in the floral bud samples. Overall, our results suggested that the unigenes specific expressed in the male or hermaphroditic flower might be potential candidates to be associated with the regulation of flower formation in andromonoecious *Taihangia*.

### Differential expressed genes (DEGs) analysis

**Fig. 3:**
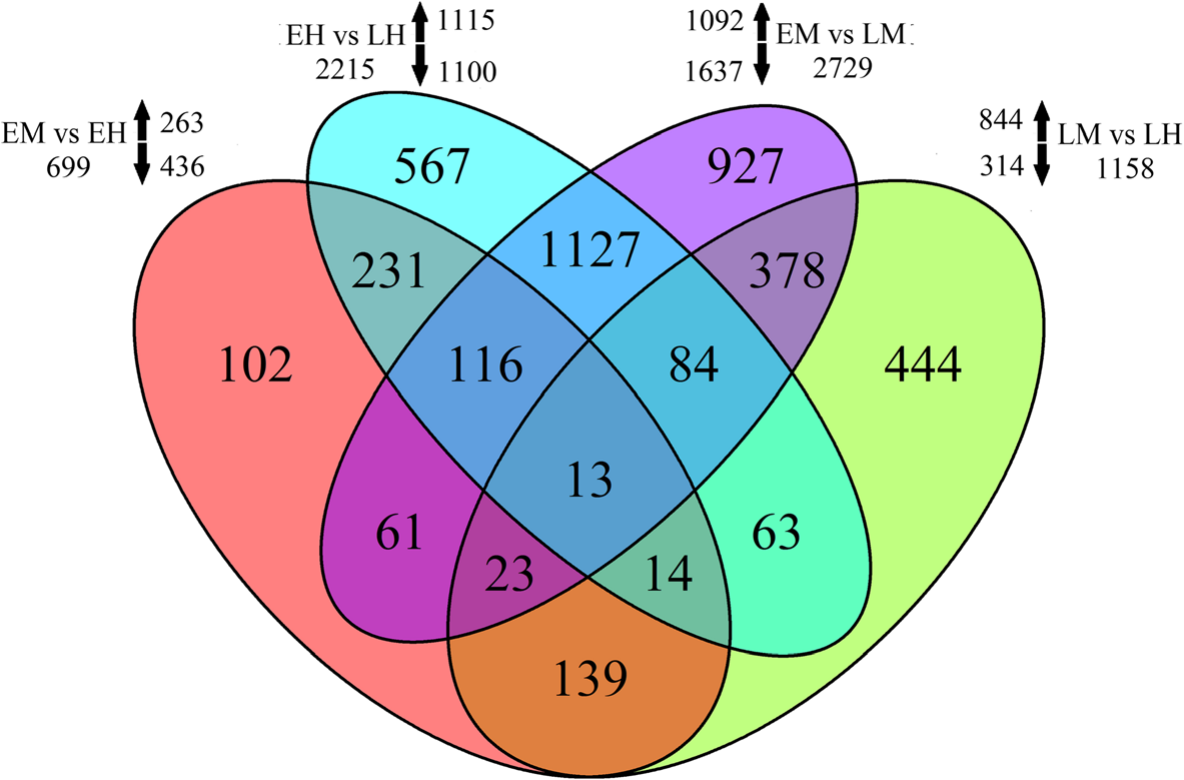
Identification of Differentially expressed genes (DEGs) in *Taihangia*. Venn diagram of the number of DEGs based on four comparisons of EM vs EH, LM vs LH, EM vs LM, and EH vs LH. Total DEG numbers in each of comparison were given outside the *circles*, and *arrows* indicate up- or down-regulation

Based on the four comparisons of EM vs EH, LM vs LH, EM vs LM, and EH vs LH, we identified a total of 4,289 differential expression genes (DEGs). Among them, 1,668 DEGs between gender types (EM + LM vs EH + LH) and 3,604 DEGs between developmental stages (EM + EH vs EH + LH) were detected, indicating that majority of DEGs occurred among different developmental stages (Fig. 3). In order to identify related genes that involved in the regulation of flower formation, 1,668 DEGs between male and hermaphroditic genders were further analyzed. At the early developmental stage, we identified 263 up-regulated genes and 436 down-regulated genes expressed in EH. On the other hand, 844 up-regulated genes and 314 down-regulated genes were detected in LH at the late developmental stage. Interestingly, 180 DEGs, including 162 hermaphroditic-biased and 18 male-biased unigenes, were differentially expressed in both early and late developmental stages, indicating that the gene expression pattern of the DEGs was maintained from the floral bud to the mature flower across developmental stages.

**Fig. 4:**
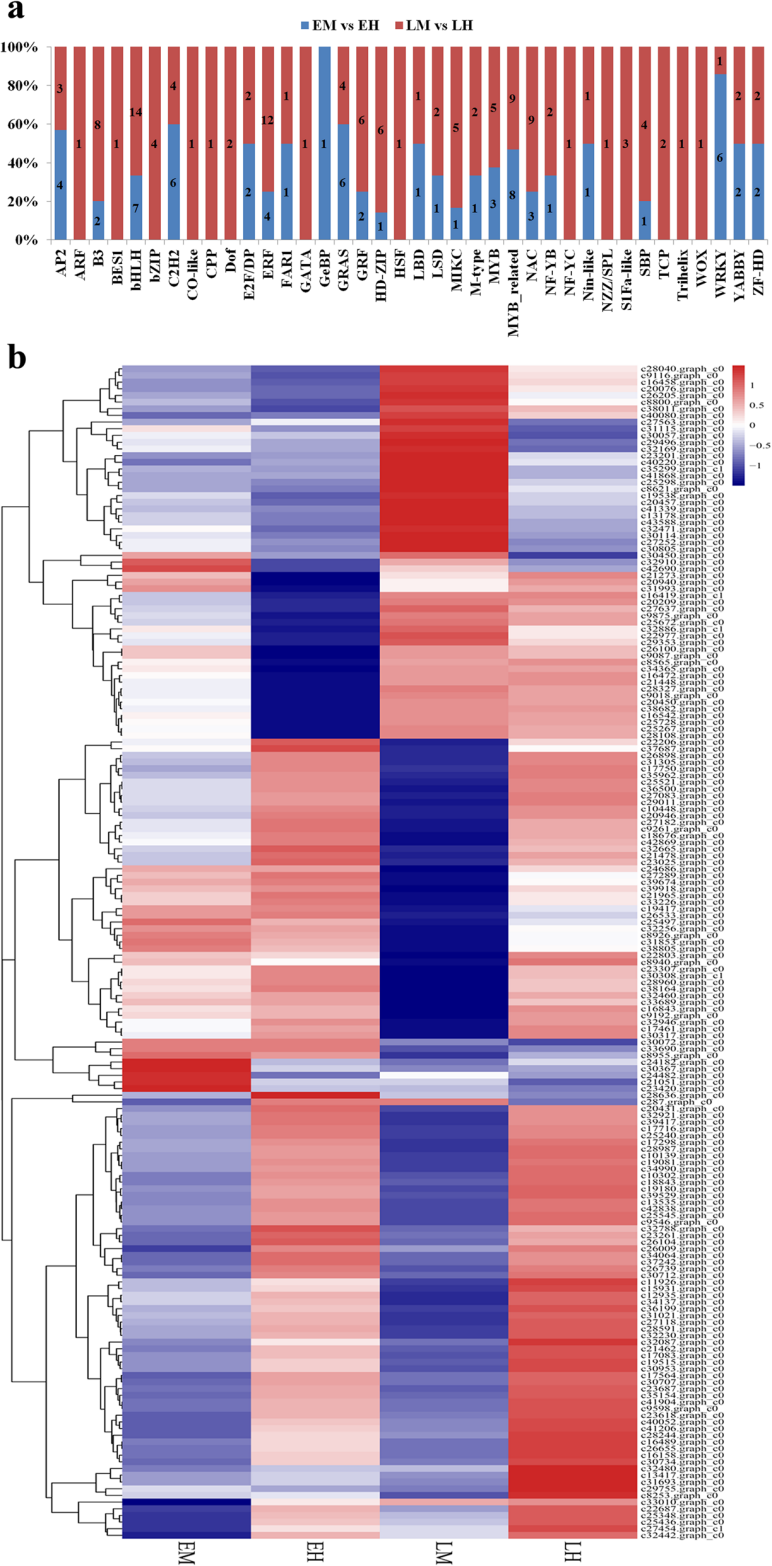
The differential expressed transcription factors between the male and hermaphroditic flowers. **a** the number of DEGs belonging to different transcription factor families detected in two comparisons of EM vs EH and LM vs LH; (**b**). Expression pattern of differential expressed TFs between the male and hermaphroditic flowers

Among the 1,661 TFs identified in this study, 176 TF genes were differentially expressed between male and hermaphroditic flowers. These TF genes fall into 38 TF families classified by the PlantTFDB 4.0, with the bHLH family (17) representing the largest amount of differentially expressed TFs, followed by ERF (15), MYB related (14), WRKY (14), and NAC (12). TF genes showed different expression patterns in male and hermaphroditic flowers during flower development (Fig. 4a), suggesting possibly different roles of TFs in the early and late developmental stages. At the early developmental stage, 67 putative TF genes belonging to 24 TF families were differentially expressed in comparison EM vs EH. The 33 TF genes belonging to 16 TF families, such as bHLH, MYB related, ZF-HD, NAC, and ERF, were significantly upregulated, while the remaining 34 TF genes belonging to 18 TF families (eg. GRF, MYB, C2H2, and AP2) were downregulated in EM. In the late developmental stage, the identified 135 putative TF genes were classified into 37 TF families based on the comparison LM vs LH. Of them, 105 TF genes were detected with significantly upregulated expression in LH. These TF genes might be important regulators contributing to the establishment and development of male and hermaphroditic flower within the andromonoecious system. The expressed profiles of TFs derived from DEGs were shown as Fig. 4b.

In summary, our results showed that the number of DEGs between male and hermaphroditic flowers remarkably increased alongside the flower development, and quite different DEG profiles were observed between comparisons EM vs EH and LM vs LH. Given that the morphogenesis of the *Taihangia* uni- and bisexual flowers occurred in the early flower developmental stages, DEGs between EM and EH were expected to be involved in sex differentiation, while DEGs between LM and LH were probably associated with floral organ development and reproduction.

### GO and pathway analyses of DEGs

**Fig. 5:**
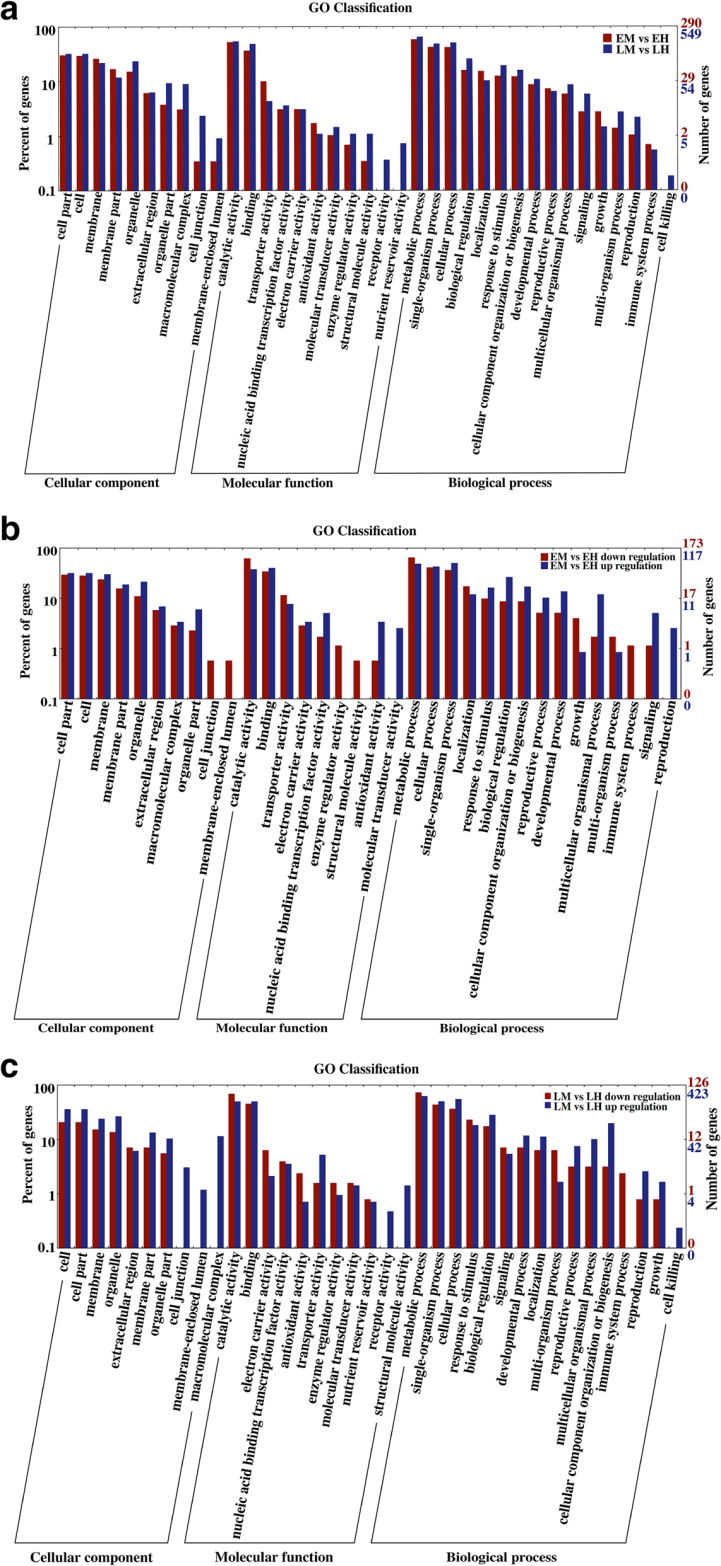
GO classifications of DEGs between the male and hermaphroditic flowers in different developmental stages. DEGs are grouped to the secondary classification of hierarchical GO terms. **a** GO classification of DEGs in two comparisons of EM vs EH and LM vs LH at early and late developmental stages; (**b**). GO classification of upregulated and downregulated DEGs in comparison EM vs EH at early developmental stage; (**c**). GO classification of upregulated and downregulated DEGs in comparison LM vs LH at late developmental stage

GO functional classifications of the DEGs between male and hermaphroditic samples were conducted by using the WEGO website. Of the identified 1668 DEGs between gender types (EM + LM vs EH + LH), 740 DEGs were classified into 37 GO subterms. In the cellular components category, the cell (GO: 0005623), cell part (GO: 0044464), membrane (GO: 0016020), and organelle (GO: 0043226), were the most abundant subterms. The catalytic activity (GO: 0003824) and the binding (GO: 0005488) subterms were dominant in the molecular function category. For the biological process category, a high percentage of DEGs was assigned to the cellular process (GO: 0009987), metabolic process (GO: 0044237), and single-organism process (GO: 0044699) subterms (Fig. 5a).

**Fig. 6:**
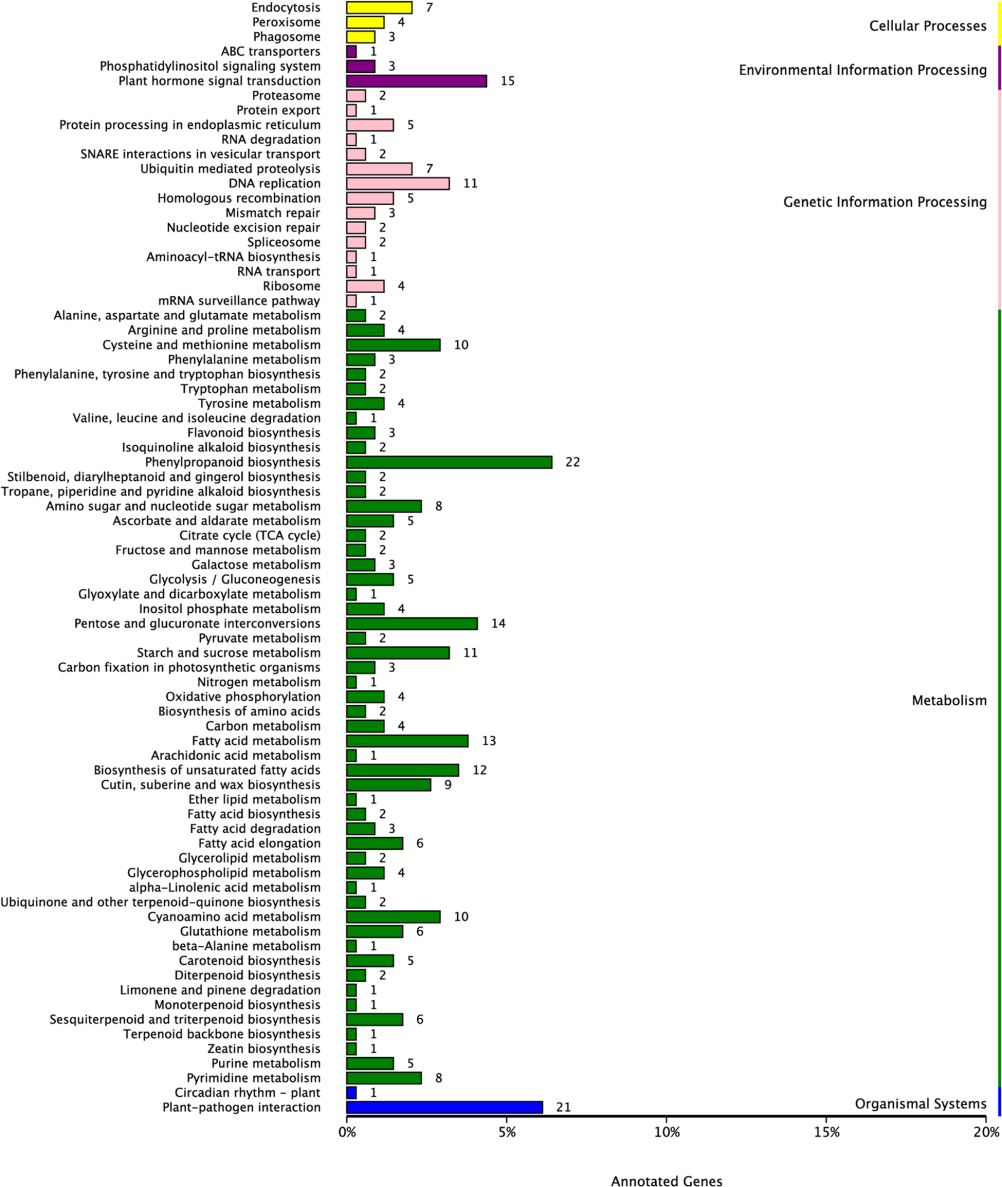
Top 50 enriched KEGG pathways of DEGs in EM vs EH and LM vs LH between male and hermaphroditic flowers. Number at the right of each column indicates the number of DEGs included in corresponding pathway. The pathways were divided into five categories: “metabolism”, “cellular processes”, “environmental information processes”, “genetic information processes”, and “organismal systems”

To better understand the regulation mechanisms of flower formation across developmental processes in *Taihangia*, we put emphasis on GO functional classifications analysis of upregulated and downregulated DEGs in comparisons EM vs EH and LM vs LH, respectively. Furthermore, GO enrichment analysis was performed by using the hypergeometric test in BinGO program with a corrected *p* value ≤ 0.05 [28]. At the early developmental stage, 290 DEGs were classified into 34 GO subterms. One hundred and seventy three upregulated genes and 117 downregulated genes in EM were classified into 32 and 29 GO subterms, respectively (Fig. 5b). Overrepresentations of GO terms in EM were found in “pollen tube development” (GO: 0048868), “regulation of pollen tube growth” (GO: 0080092), “hydrolase activity, acting on glycosyl bonds” (GO: 0016798), indicating that these genes involved in stamen development. For up-regulated DEGs in EH, most of the overrepresented GO terms were related to cell cycle, developmental process, and metabolic process, such as “regulation of macromolecule metabolic process” (GO: 0060255), “regulation of cell cycle” (GO: 0051726), “anatomical structure development” (GO: 0048856), “integral component of membrane” (GO: 0016021), and “regulation of transcription, DNA-templated” (GO: 0006355). In the late developmental stage, 38 GO subterms were found for 549 DEGs in comparison LM vs LH. One hundred and twenty six upregulated genes and 423 downregulated genes were classified into 32 and 37 GO subterms, respectively (Fig. 5c). The overrepresentations of GO terms in LM were observed in “oxidoreductase activity” (GO: 0016491) while overrepresentations of GO terms in LH mainly included “cell cycle process” (GO: 0022402), “DNA methylation” (GO: 0006306), “flower development” (GO: 0009908), and “DNA binding” (GO: 0003677). In particular, a large number of GO terms, such as “regulation of cell cycle”, “regulation of transcription, DNA-templated”, and “regulation of developmental process” (GO: 0050793) overrepresented in early developmental stages were also enriched at late developmental stages. The GO enrichment analysis results are presented in Additional file 10.

To further investigate the biochemical pathways of these DEGs, we mapped the 1,668 DEGs to terms in the KEGG database. Among them, 219 genes had a KO ID and could be categorized into 78 pathways, of which “Phenylpropanoid biosynthesis” (22), “Plant-pathogen interaction” (21), “Plant hormone signal transduction” (15), “Pentose and glucuronate interconversions” (14), “Fatty acid metabolism” (13), and “unsaturated fatty acid metabolism” (12) had the most DEG numbers (Fig 6, Additional file 11). In addition, we conducted an enrichment analysis with the KEGG pathway, which assigned the DEGs to 9 pathways such as biosynthesis of unsaturated fatty acids, phenylpropanoid biosynthesis, and pentose and glucuronate interconversions (corrected *p* value ≤ 0.05). In particular, two pathways “unsaturated fatty acid metabolism” (12) and “DNA replication” (11) were highly represented, respectively, which were consistent with the GO enrichment analysis.

**Fig. 7:**
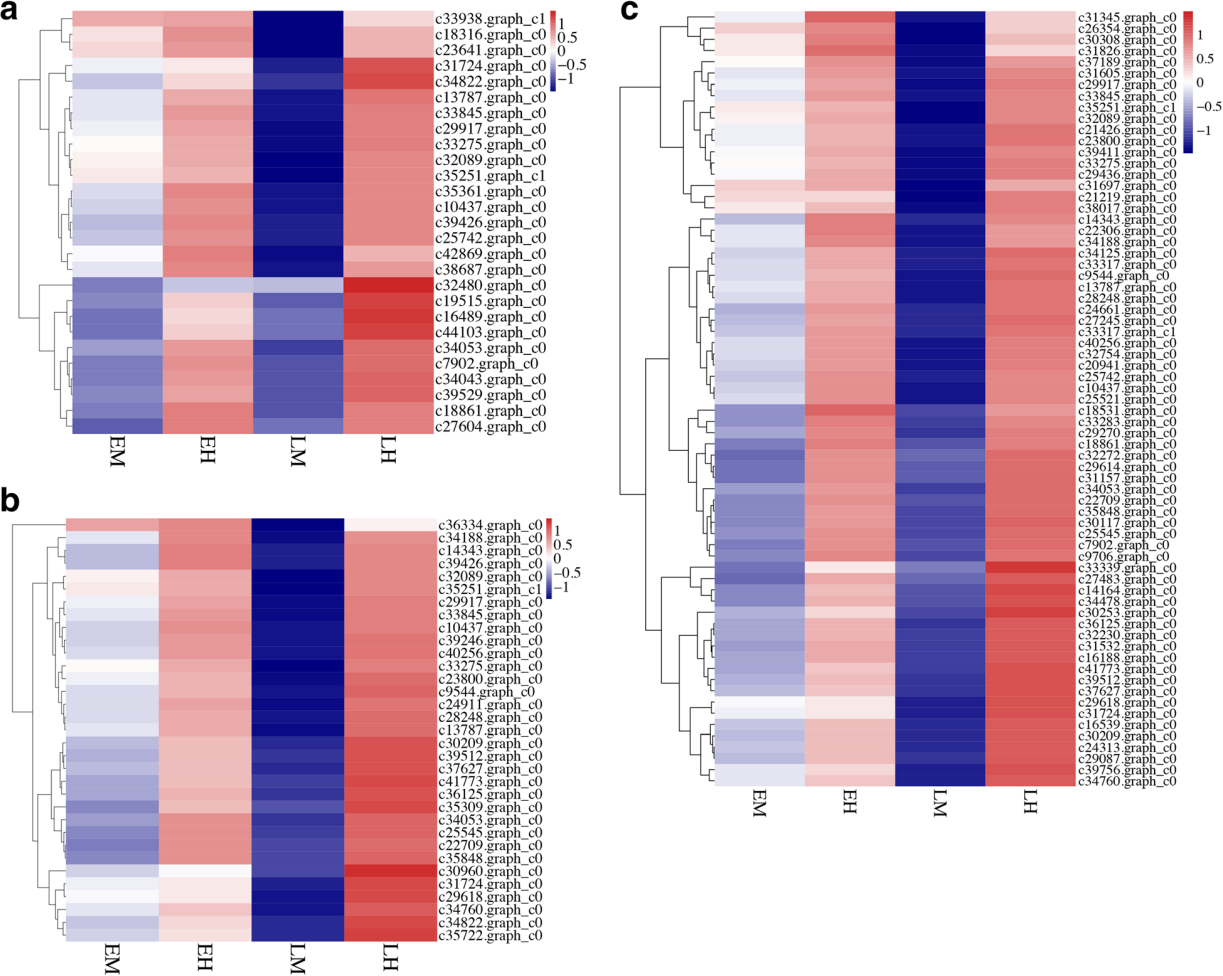
Expression pattern of functional DEGs involved in sex differentiation and flower organ development. **a** Flower development; (**b**) Epigenetic processes; (**c**) Cell proliferation. The *bar* represents the scale of relative expression levels of DEGs. *Red* means high expression and *blue* means low expression

GO and KEGG pathway analysis revealed that DEGs between the two genders were associated with a wide range of functions, and participated in many biological processes, such as cell proliferation, chromatin modification, and flower development, suggesting that the formation of uni- and bisexual flowers might be controlled by many genes involved in numerous biological processes. Moreover, the identified GO subterms and KEGG pathways could provide further information on the molecular mechanism of sex differentiation in andromonoecious *Taihangia*.

### DEGs involved in regulation of *taihangia* male and hermaphroditic flower formation

#### Flower development

Based on DEG analysis and gene expression profiles of male and hermaphroditic flowers, we identified a serial of DEGs involved in flower development (Fig. 7a). Among them, several genes related to floral organ development, such as MADS-box, WUSCHEL (WUS), YABBY1 (YAB1), and LRR-protein kinase BAM3, were up-regulated in hermaphroditic flowers. Particularly, four differentially expressed MADS-box genes were found in the AG, B-sister (Bs), and MIKC* subfamilies. Among them, both TruMADS4 (AG) and TruMADS8 (FBP24) were up-regulated in hermaphroditic flowers at late developmental stages. TruMADS20 (STK), belonging to D-class functional genes, showed dramatically higher expression in hermaphroditic than in male flowers throughout floral development. Our results indicated that these MADS-box genes preferably expressed in LH were likely associated with gynoecium development in hermaphroditic flowers. Conversely, TruMADS22, belonging to MIKC* subfamilies, was upregulated in EM at early developmental stages, suggesting that the MADS-box TF probably involved in stamen development.

#### Epigenetic process

Epigenetic mechanisms, including DNA methylation, histone modifications, chromatin conformation, and RNA interference, play essential roles in modulating gene expression without altering the DNA sequence. In this study, a number of DEGs involved in DNA methylation and histone modification were identified in *Taihangia* flower at late developmental stage (Fig. 7b). Several genes, including three DNA methyltransferase CMT3, MET, and DRM2, involved in DNA methylation process showed differentially expression between male and hermaphroditic flowers. On the other hand, histone modifications related genes, such as histone-lysine N-methyltransferase ATXR6, ASHR3, and SUVR4, were differentially expressed in comparison LM vs LH at late developmental stages. No significant differences were observed in the expression levels of epigenetic related genes between male and hermaphroditic flowers at the early developmental stages, while all of them were significantly upregulated in LH at late developmental stages. Notably, one homologous of AGO 16 gene involved in RNA silencing and RNA-directed DNA methylation (RdDM) pathway exhibited upregulated expression patterns in LH at late developmental stages. Our finding indicated that epigenetic processes may extensively occur in hermaphroditic flowers, especially at late developmental stages.

#### Cell cycle process

A large number of DEGs involved in cell cycle process, such as DNA replication, cell division, and regulation of cell cycle, were identified based on transcriptome data, and these DEGs showed significantly increased expression in hermaphroditic flowers at late developmental stage (Fig. 7c). A number of DEGs, including mitotic spindle checkpoint protein MAD2-like, ATP-dependent DNA helicase Q-like, DNA topoisomerase 3-alpha-like, and mitotic checkpoint serine/threonine-protein kinase BUB1 beta-like were assigned into DNA replication functional group. Meanwhile, it was found that majority of DEGs, belonging to cell division functional group, were down-regulated in LM at late developmental stage, including 65-kDa microtubule-associated protein, G2/mitotic-specific cyclin-1-like, condensin complex subunit 2-like, DNA replication licensing factor mcm2-like, and DNA replication licensing factor MCM3. Similarly, several DEGs involved in cell cycle group also showed down regulated expression in male unisexual flowers, such as microtubule-associated protein RP/EB family member 1C, DNA cross-link repair protein SNM1-like, protein FIZZY-RELATED 3-like, and DNA cross-link repair protein SNM1-like. In particular, many DEGs, including G2/mitotic-specific cyclin-1-like, protein FIZZY-RELATED 3-like, mitotic spindle checkpoint protein MAD2-like, mitotic checkpoint serine/threonine-protein kinase BUB1 beta-like, cyclin-A1-1-like and cyclin-D3-1-like, showed significantly upregulated expression in both EH and LH across the developmental stages. Overall, our results indicated that the cell cycle process was probably severely disturbed in male unisexual flowers.

#### Biosynthesis of unsaturated fatty acids

Unsaturated fatty acids are considered to be essential membrane components, and play key roles in many cellular events. The synthesis of unsaturated fatty acid is a complicated process, requiring for involvement of a series of enzymes, such as palmitoyl-monogalactosyldiacylglycerol Δ7 desaturase (FAD5) and acyl-[acyl-carrier-protein] desaturase 6 (ACP 6). In the present study, several DEGs encoding several key enzymes invovled in the biosynthesis of unsaturated fatty acids pathway, including six FAD5, three very-long-chain 3-oxoacyl-CoA reductase 1-like genes (ACPR), and one ACP6 genes, were found to be significantly downregulated in male flowers, indicating that biosynthesis of unsaturated fatty acids was likely restricted in male flowers.

### Validation of unigenes and gene expression profiling using RT-qPCR

**Fig. 8:**
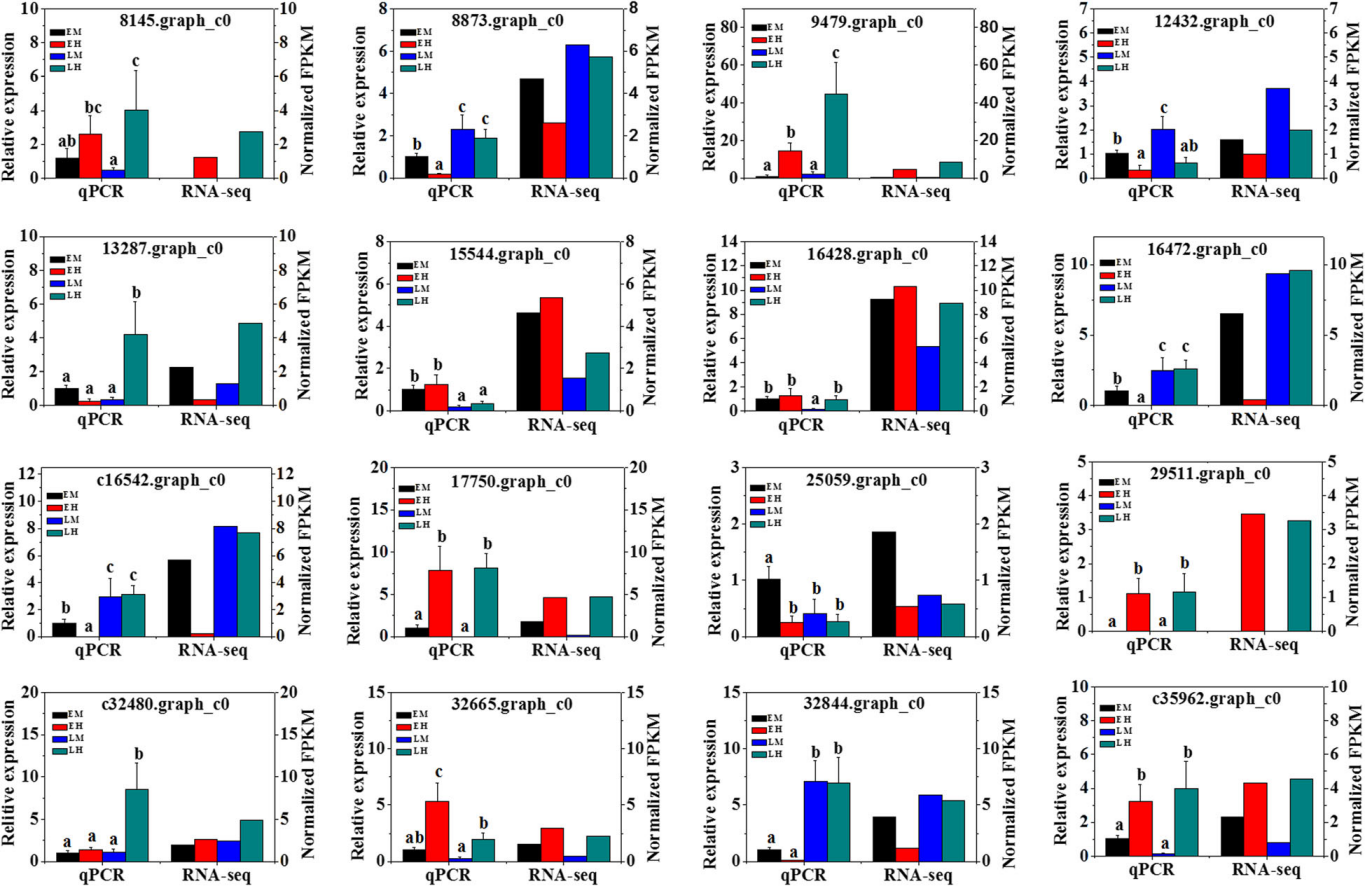
Validation of 16 randomly selected differentially expressed genes (DEGs) derived from RNA-seq using Real-time quantitative RT-PCR. Quantitative gene expression data represented as the mean ± SD are shown on the left. The EF1α and UBQ were used as reference genes. Statistically significant differences of relative expression derived from one-way ANOVA at the level of *P* < 0.05 were marked as different lowercase letters. The normalized FPKM, transformed by log_2_ (1+ FPKM), were indicated on the right

To confirm the gene expression patterns identified by the RNA-seq data, the transcript levels of 16 random selected DEGs were examined by quantitative PCR. We performed qRT-PCR using these 16 genes with 3 biological replicates from each group (EM, EH, LM, and LH) (n = 12). The detailed information of the selected DEGs, IDs and primer pairs used in this study were shown in Additional file 12: Table S4. It was found that all of 16 selected DEGs were successfully amplified with single bands with the expected sizes, and the patterns of gene expression detected by RT-qPCR were consistent with those from RNA-Seq data (Fig 8). Therefore, RT-qPCR experiments confirmed that the DEGs obtained from the assembled transcriptome were accurate and gene expression profiles from RNA-Seq data were reliable.

## Discussion

### *Taihangia* transcriptome profiles

As an andromonoecious plant species, *Taihangia* provides an excellent model system within a uniform genetic background for the study of sex differentiation and establishment of unisexual flowers. In this study, we conducted *de novo* transcriptome sequencing to compare transcriptional profiles of the male and hermaphroditic flowers by using the Illumina RNA-Seq method. To the best of our knowledge, this is the first report of transcriptome data in this rare cliff plant species with the andromonoecious sexual system. A total of approximately 84,596,426 qualified Illumina reads (Q30 > 91%) were obtained from four floral sequencing libraries and then assembled into 59,064 unigenes. Of the assembled unigenes, 25,231 unigenes (42.33%) were annotated in the public database, while the function of other 33,833 assembled unigenes (57.67%) could not be identified. Given that the genomic and transcriptomic information is limited for *Taihangia*, the lineage-specific genes associated with the unique floral phenotype may not be contained in function annotation, implying that potential novel genes were discovered from our transcriptome data. By comparative analysis of male and hermaphroditic transcriptome data, we identified a large number of candidate DEGs involved in various biological processes, which is likely associated with the regulation of male and hermaphroditic flower formation. The transcriptome data provide an important resource for the understanding of specific biological processes involved in sex differentiation and facilitating gene discovery in the future studies in andromonoecious *Taihangia*.

### Floral organ identity-related MADS-box genes may be crucial for flower organ formation

The MADS-box transcription factors, initially identified as floral homeotic genes, are the major components in the well-known ‘ABCDE’ model that describes their roles in the determination of floral organ identity [34]. Based on *Taihangia* transcriptome data, we identified 25 MADS-box TFs, including several MADS proteins in each putative functional group described in ABCDE model. According to the “ABCDE” model [35], similar levels of expression of class A-, B-, and E-class MADS-box genes between male and hermaphroditic genders were expected, while class C- and D- MADS genes should be upregulated expressed in the hermaphroditic flowers. In the present study, most of MADS-box TFs including A-, B- and E-class of genes did not show differential expression between male and hermaphroditic flowers. As expected, two MADS-box TFs, TruAG (C-class) and TruSTK (D-class), showed upregulated expression in hermaphroditic flowers. However, it was noteworthy that upregulated expression of TruAG was not found in EH but in LH, suggesting that its expression pattern appears to follow the arrest of pistil development in male flowers. A number of studies have found that the MADS box genes did not play a part in sex determination in some dioecious and monoecious species that achieve unisexuality by organ abortion [36, 37]. In *Taihangia*, pistils are initiated and then arrest in unisexual male flower, the molecular mechanism of sex determination must be acting downstream of organ identity. Thus, the transcriptional profile of several MADS-box TFs more expressed in hermaphroditic flowers might be the consequence rather than the cause of sex differentiation in andromonoecious *Taihangia*, and the MADS-box TFs were related to pistil development in hermaphroditic flowers.

### Epigenetic-related genes are likely associated with reproduction in hermaphroditic flower

Epigenetic processes such as DNA methylation, histone modification, and small RNA, play important roles in the regulation of gene expression during flower development [38]. As key enzymes involved in DNA methylation processes, DNA methyltransferases and histone H3K9 methyltransferase are crucial for establishment and maintenance of DNA methylation [39]. In our study, three DNA methyltransferases (MET1, DRM2, and CTM3) and three histone H3K9 methyltransferase (ASHR3, ATXR6, and SUVH4) showed preferable expression in LH, indicating their possible roles in *Taihangia* reproduction at the late developmental stage. In addition, several genes, such as Argonaute 16 (AGO16), suppressor of gene silencing 3 (SGS 3, c26995.graph_c0), RNA-dependent RNA polymerase 3 (RDR3, c36802.graph_c0), Dicer homolog 3a-like (DCL3, c35108.graph_c0), and RPD1 (c38350.graph_c0), possibly involved in RNA-induced silencing and RNA-directed DNA methylation (RdDM) pathways were identified based on *Taihangia* transcriptome data. Particularly, AGO 16 showed approximately three folds higher expression in LH than in LM at late developmental stage. As key regulators, AGO proteins and DCL3, along with RDR, are crucial for the epigenetic silencing [40]. Based on gene expression analysis, three genes, DCL, RDR, and RPD, showed more than two times higher expressions in LH than in LM*,* indicating that extensive small RNA related processes possibly occurred in *Taihangia* hermaphroditic flower at developmental stage. Currently, increasing evidences have supported that epigenetic processes played important roles in regulation of plant reproductive development [41–44]. For example, Galla et al. [41] reported that small RNA generation related genes, including DCL3, AGO, SGS3, and RDR, were involved in plant reproduction, such as cell identity and fate in the ovule, in *Hypericum perforatum*. The upregulated expressions of these epigenetic-related genes in *Taihangia* hermaphroditic flowers, especially at the late developmental stages, may indicate that the epigenetic regulation played important roles in plant reproduction, such as female gametophyte formation. The roles of epigenetic processes in *T. rupestris* are still unclear, and future studies are needed to investigate the roles of associated genes involved in epigenetic processes.

### Disturbance of cell cycle may contribute to pistil abortion in male flower

During flower development, arrest of pistil or stamen development is a normal phenomenon in monoecious and dioecious plant [45–47]. Several biological processes, such as programmed cell death (PCD) and cessation of the cell division, are critical in establishment of unisexual flower [48]. Based on *Taihangia* RNA-seq data, a great number of DEGs involved in cell cycle, DNA replication, and cell division were significantly downregulated in male flowers over the developmental stages, indicating that these biological processes played important roles in arrest of pistil development in *Taihangia* male flowers. For many plant species, PCD is essential to many aspects of plant morphogenesis, and it is a common process for the developmental arrest of reproductive organs in unisexual flowers [45, 49, 50]. However, we did not found that PCD involved in pistil abortion in *Taihangia* male flowers based on transcriptome data. It has been reported that developmental arrest of sterile sex organs and the subsequent unisexuality of flowers could resulted from a cessation of cell division [46]. Similarly, down-regulated expressions of related genes involved in cell division probably led to restriction of cell proliferation, which may be closely associated with the arrest of pistil development in *Taihangia* unisexual male flower.

As primary regulatory genes, cyclins play critical roles in controlling cell cycle progression, including DNA replication, the G2/M transition, and mitosis [51]. In this study, several cyclin genes (CYCA1, CYCA2, CYCA3, and CYCD3) related to regulation of cell cycles were found to be uniformly down-regulated in unisexual male flowers. Particularly, cyclin-A1-1-like and cyclin-D3-1-like significantly decreased expression level in EM and LM across developmental stages, indicating that there was a potential mechanistic link between the regulation of cell cycle and the establishment of unisexual flowers in *Taihangia*. During flower development, cyclin-A1and cyclin-D3 were considered as the core regulatory genes involved in regulation of flower primordia information and meristem activity [52]. For example, cyclin-D3 could define distinct developmental zones, acting as a developmental boundary between lateral organs, in the shoot apical meristems by controlling the balance between cell division and cell differentiation [51]. In *Taihangia* unisexual male flowers, downregulated expression of CYCA1and CYCD3 probably resulted in the disturbance of cell cycle in gynoecium primordia, and subsequently determined arrest of pistil development. These results suggested that the core regulatory genes involved in cell cycle progression probably contribute to pistil abortion in *Taihangia* unisexual flowers by regulation of the cell cycle.

### Cold-responsive genes are likely to affect the formation of unisexual flowers

It is well known that the establishment of unisexual flower is influenced by environmental factors such as temperature, light intensity, photoperiod, and nutritional conditions via specialized gene expression [53–55]. For andromonoecious *Taihangia*, temperature during the initiation of floral primordia is crucial for determining morphogenesis of gender type, and generation of unisexual male flowers is attributed to pistil abortion under low temperature [9]. To deal with adverse cold stress, plants have developed versatile defense mechanisms to acquire cold tolerance by adjustments in molecular, and physiological, and developmental processes [56]. For example, increase of membranes’ unsaturated fatty acid content could promote the stabilization of plasma and chloroplast membranes, and thus improve resistance to cold stress [57]. As key enzymes involved in biosynthesis of unsaturated fatty acid, FAD7, ACPR, and ACP 6 were dramatically down-regulated in EM, indicating that restriction of polyunsaturated fatty acids biosynthesis probably occurred in *Taihangia* male flowers. Under low temperature condition, a number of cold-regulated genes such as LEA family genes could be induced in higher plants [54]. For instance, LEA proteins are thought to play a role in membrane stabilization by interacting with membrane surfaces or with water molecules under cold stresses [58]. Based on transcriptome data, a number of cold-resistance genes, including LEA Dc3, LEA B19.3, ascorbate oxidase, and peroxidase, were significantly down-regulated in male flowers. These results agreed well with a recent study on comparative transcriptome analysis between male and hermaphroditic flowers in Japanese apricot by Shi et al. [6], arguing that reduction of cold-resistance gene expression in male flowers is likely associated with pistil abortion under low temperature. Taken together, these results suggested that down-regulated expression of cold-responsive genes at the early stage is likely to affect the formation of unisexual male flowers in andromonoecious *Taihangia*.

### Candidate TFs as important regulators in establishment of male and hermaphroditic flowers

In plant, TF genes play an important role in many aspects of plant growth and development [59]. In the present study, we identified a large number of TF genes showing significantly differential expression between male and hermaphroditic flowers. These TFs are expected to be involved in the regulation of formation of the male and hermaphroditic flowers in *Taihangia*. Of these identified TFs, most stress-responsive TFs, such as NAC, WRKY, bHLH, GRAS, and ERF showed increased expression in male flowers, implying that male flowers probably suffered more severe injuries than hermaphroditic flowers. Majority of upregulated TFs in bisexual flowers, including WOX, YAB, AP2, and C2H2, were associated with flower organ development. For example, WUSCHEL (WUS), a number of WOX TF genes, is known to maintain the stem cell activity in the floral meristems [60]. After floral induction, WUS can induce AG expression and promote the up-regulation of AG in developing flowers [61]. In our study, higher expression of one homolog of WUS was not observed in hermaphroditic flowers at early developmental stages. Similarly, Tru*AG* showed no difference in expression between EM and EH, suggesting that a possible different regulatory mechanism occurred in *Taihangia* flower development. The YABBY gene family is involved in adaxial–abaxial polarity in lateral organs, and participates in a diverse range of processes such as floral patterning, organ growth, and maintenance of meristem organization [62, 63]. One homologous of YABBY1 genes identified in this study was upregulated in both EH and LH, indicating that YABBY TFs were closely associated with the formation of bisexual flowers. Interestingly, five genes encoding growth-regulating factor (GRF) were detected and showed uniformly increased expression in hermaphroditic flowers. GRFs are plant-specific transcription factors, which play important roles in the control of cell proliferation via cell cycle regulation and central developmental processes including flower formation [64, 65]. Recently, Liang, et al. reported that dramatically reduced expression levels of GRF caused pistil abnormalities and suggested an important role of GRF in controlling carpel number and pistil development [66]. Our result was consistent with the finding in *Arabidopsis*, and confirmed that disturbance of cell proliferation in male flowers contributed to the pistil abortion.

## Conclusion

*De novo* transcriptome sequencing of andromonoecious *Taihangia* was performed by using the Illumina paired-end sequencing technology in the present study. In total, 59,064 unigenes were obtained from the male and hermaphroditic flowers covering early and late developmental stages, and 25,231 unique sequences were annotated in the public database. Focusing on the regulatory mechanism of flower formation in andromonoecious *Taihangia*, we identified 1,668 DEGs between male and hermaphroditic flowers. GO and pathway analysis showed that these DEGs involved in a wide range of biological processes, such as flower development and reproduction, cell cycle, epigenetic processes, and transcriptional regulation, and provided numerous candidate genes possible related to sex differentiation for future functional analysis. The transcriptome data of *Taihangia* could be helpful to improve understanding of the molecular mechanisms in sex differentiation and unisexual flower establishment, and would provide theoretical basis for the regulation of flower formation in this andromonoecious plant species.

## Additional files

Additional file 1: Figure S1. Male and hermaphroditic flowers produced within the same individual of *Taihangia* on the cliff face. (PDF 671 kb)
Additional file 2: Figure S2. Length distribution of the unigene sequences. (TIF 2980 kb)
Additional file 3: Table S1. Summary for the alignment of reads to unigene libraries. (DOCX 14 kb)
Additional file 4: Table S2. Validation of assembled unigenes by using Sanger sequencing. (DOCX 17 kb)
Additional file 5 Figure S3. Characteristics of the homology search of *Taihangia* unigenes against the NR database. (PDF 136 kb)
Additional file 6: Functional GO, COG, and KEGG pathway annotation and classification of the *Taihangia* unigenes. (XLSX 3235 kb)
Additional file 7: Identification of transcription factors against PlnTFDB database. (XLSX 226 kb)
Additional file 8: Table S3.
*Taihangia* MADS-box gene names and attributes. (DOCX 17 kb)
Additional file 9: Figure S4. Venn diagram of the number of specific and shared unigenes in EM, EH, LM, and LH samples. (TIF 1967 kb)
Additional file 10: Over-representative GO terms of DEGs in EM vs EH, and LM vs LH, respectively. (XLSX 74 kb)
Additional file 11: Pathway classification and enrichment analysis of the DEGs between the male and hermaphroditic flowers. (XLSX 25 kb)
Additional file 12: Table S4. The selected DEGs, IDs, and primer pairs for RT-qPCR. (DOCX 18 kb)

## Abbreviations

COG: Clusters of Orthologous Group
DEG: Differentially expressed gene
FPKM: Fragments per kilobase of exon per Million fragments mapped
GO: Gene Ontology
KEGG: Kyoto Encyclopedia of Genes and Genomes
qRT-PCR: quantitative reverse transcription polymerase chain reaction
RdDM: RNA direct DNA methylation
RNA-Seq: Ribonucleic acid sequencing
*Taihangia rupestris*: *Taihangia*
TF: Transcription factor
