# Supplementary figures and images for: De novo sequencing and comparative transcriptome analysis of the male and hermaphroditic flowers provide insights into the regulation of flower formation in andromonoecious taihangia rupestris
Source: BMC Plant Biol. 2017 Feb 28;17:54. doi: 10.1186/s12870-017-0990-x (PMC5329940; doi:10.1186/s12870-017-0990-x)

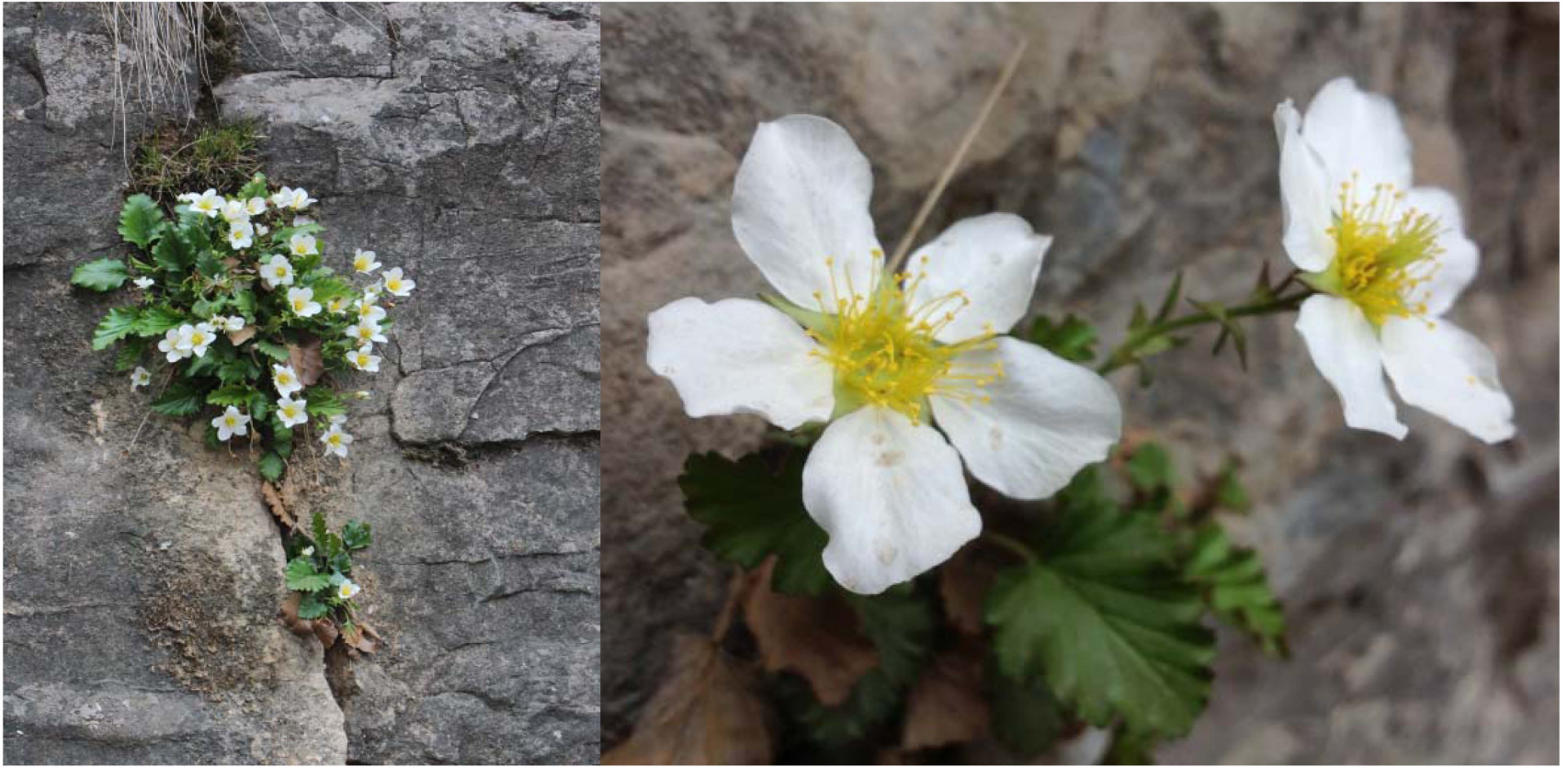

Figure S1. Male and hermaphroditic flowers within andromonoecious *Taihangia* on the cliff face.

Supplement: Additional file 1: Figure S1. — Male and hermaphroditic flowers produced within the same individual of Taihangia on the cliff face. (PDF 671 kb) [file 12870_2017_990_MOESM1_ESM.pdf]

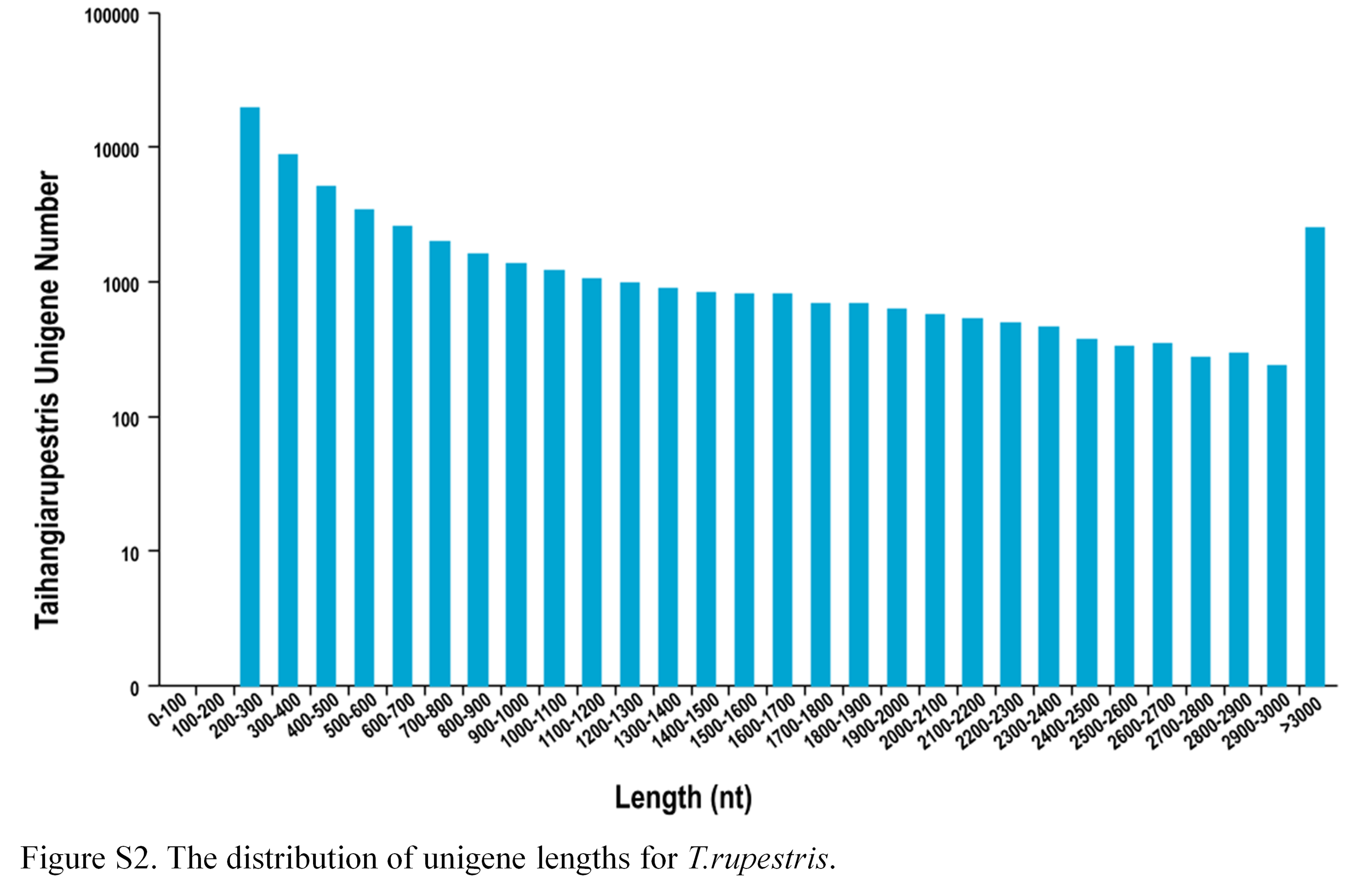

Supplement: Additional file 2: Figure S2. — Length distribution of the unigene sequences. (TIF 2980 kb) [file 12870_2017_990_MOESM2_ESM.tif]

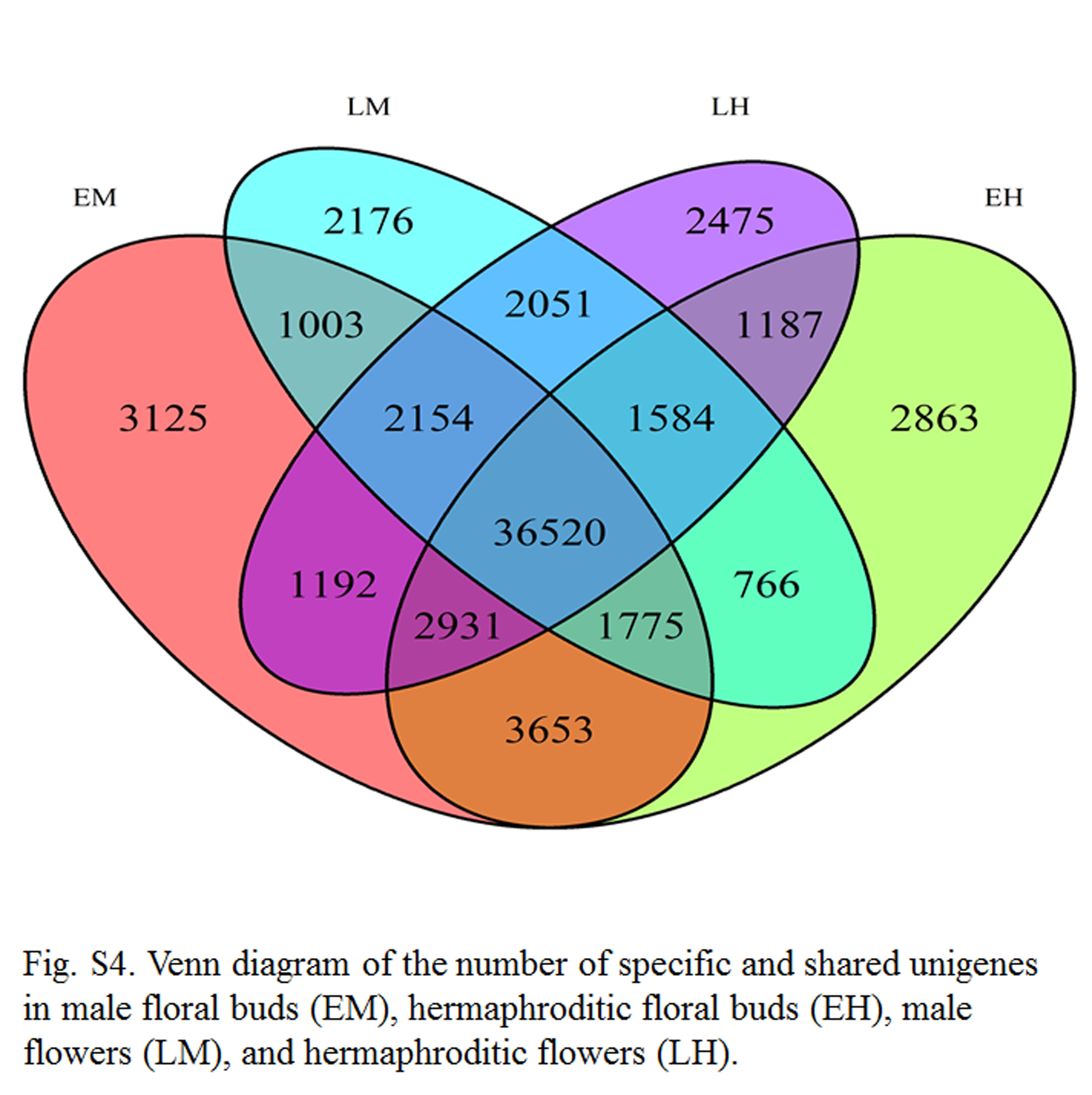

Supplement: Additional file 9: Figure S4. — Venn diagram of the number of specific and shared unigenes in EM, EH, LM, and LH samples. (TIF 1967 kb) [file 12870_2017_990_MOESM9_ESM.tif]
