# Supplementary material for: De novo sequencing and comparative transcriptome analysis of the male and hermaphroditic flowers provide insights into the regulation of flower formation in andromonoecious taihangia rupestris
Source: BMC Plant Biol. 2017 Feb 28;17:54. doi: 10.1186/s12870-017-0990-x (PMC5329940; doi:10.1186/s12870-017-0990-x)
Supplement: Additional file 3: Table S1. — Summary for the alignment of reads to unigene libraries. (DOCX 14 kb) [file 12870_2017_990_MOESM3_ESM.docx]

Table S1. Summary for the alignment of reads to unigene libraries

| Sample ID | Total reads | Mapped reads | Unique mapped Reads | Multiple mapped Reads |
| --- | --- | --- | --- | --- |
| EM | 21,136,736 | 17,845,018(84.43%) | 11,937,405(66.89%) | 5,907,613(33.11%) |
| EH | 20,963,888 | 17,640,460(84.15%) | 11,970,934(67.86%) | 5,669,526(32.14%) |
| LM | 21,096,120 | 17,919,516(84.94%) | 12,051,629(67.25%) | 5,867,887(32.75%) |
| LH | 21,399,682 | 18,054,634(84.37%) | 12,135,925(67.22%) | 5,918,709(32.78%) |
