# Supplementary material for: De novo sequencing and comparative transcriptome analysis of the male and hermaphroditic flowers provide insights into the regulation of flower formation in andromonoecious taihangia rupestris
Source: BMC Plant Biol. 2017 Feb 28;17:54. doi: 10.1186/s12870-017-0990-x (PMC5329940; doi:10.1186/s12870-017-0990-x)
Supplement: Additional file 4: Table S2. — Validation of assembled unigenes by using Sanger sequencing. (DOCX 17 kb) [file 12870_2017_990_MOESM4_ESM.docx]

Table S2. Validation of assembled unigenes by using Sanger sequencing

| ID | Primer (5’ – 3’) | Product (bp) | E value | Bit score | Identical |
| --- | --- | --- | --- | --- | --- |
| c29239_c0 | F: GGAGACCAGCCAATGAAGAAT | 213 | 2.00E-86 | 317 | (160/160) 1.00 |
|  | R:CAAGACAAAGAGGACCTACCGA |  |  |  |  |
| c9145_c0 | F: CAGCCTTACTTCCGTTGG | 208 | 3.00E-88 | 323 | (170/171) 0.99 |
|  | R: ACCCTCATTTTCACATTTCC |  |  |  |  |
| c30602_c0 | F: CCAATCAAGGTTGTCTCA | 184 | 4.00E-78 | 289 | (146/146) 1.00 |
|  | R: CATAGGTAGGAATGTCGG |  |  |  |  |
| c16607_c0 | F: CCCTGGGCAGATTGGAAACG | 245 | 4.00E-94 | 343 | (179/181) 0.98 |
|  | R: CCTCACAGCAAAGCGACCGA |  |  |  |  |
| c16542_c0 | F: GGTGAGGCAGGCAAGAAAAT | 145 | 1.00E-49 | 194 | (98/98) 1.00 |
|  | R: GTGATGAGGCGAGGAGGC |  |  |  |  |
| c38440_c0 | F: GTCTACAAGGACCACATCACT | 135 | 4.00E-43 | 172 | (94/95) 0.98 |
|  | R: CTTCTTATTTACGACACCGA |  |  |  |  |
| C38452_c0 | F: AAGCCCCACAGATACCGT | 212 | 3.00E-88 | 323 | (163/163) 1.00 |
|  | R: GTGTCCTCAAACAACCCAAC |  |  |  |  |
| c26982_c0 | F: GATTTAGGTCGCTGAGATAG | 126 | 3.00E-37 | 153 | (77/77) 1.00 |
|  | R: TCAAGGGTGAAGATGTAGTC |  |  |  |  |
| c25059.c0 | F: CCAAAGCACAGGTTTACAAG | 171 | 8.00E-73 | 272 | (137/137) 1.00 |
|  | R: AGGAGACCACCAACAATAGAC |  |  |  |  |
| c20350_c0* | F:GAAGGGTAGCGGAGACAGAA | 223 | 2.00E-86 | 317 | (175/180) 0.97 |
|  | R:ATAGCCCGTTCCTTCGTTTT |  |  |  |  |
| c20350_c0* | F:TGGGTATCACCAGAGCG | 342 | 1.00E-154 | 543 | (284/286) 0.99 |
|  | R:GTGGAGGAGCACAAACG |  |  |  |  |
| c36974_c0* | F: TAATCTAATCCCAACCCTC | 343 | 1.00E-125 | 446 | (277/293) 0.94 |
|  | R: CATTCATACCGAAATAACCT |  |  |  |  |
| c24891.c0* | F: AAGCCCTGTTGGTCATTGTT | 284 | 1e-121 | 434 | (219/219)1.00 |
|  | R: AAGCCCTCCATCTCCACTAT |  |  |  |  |
| c31334.c0* | F: ATAATAACAGCCAGAACGGAG | 271 | 8e-68 | 256 | (132/133) 0.99 |
|  | R: CGAACGAATGAAAAGGACC |  |  |  |  |
| c27945.c0* | F: ACTCGTCGTCATCCCGTCTAT | 275 | 1e-79 | 295 | (149/149) 1.00 |
|  | R: GTAAGGGTCTCGCCGTATCG |  |  |  |  |
| c37327_c2* | F: CACAGCCAAATAGTAAAGC | 291 | 5.00E-35 | 147 | (77/78) 0.98 |
|  | R: AGACGAGGAGGAAGAAGAG |  |  |  |  |
| c35356.c0* | F: CGCCAAAAGGAATCTAAGTC | 199 | 1e-68 | 258 | (153/159) 0.96 |
|  | R: AGTAGGGCAGAAGAAAAGTGA |  |  |  |  |

*Template DNA: Genomic DNA.
