# Supplementary material for: De novo sequencing and comparative transcriptome analysis of the male and hermaphroditic flowers provide insights into the regulation of flower formation in andromonoecious taihangia rupestris
Source: BMC Plant Biol. 2017 Feb 28;17:54. doi: 10.1186/s12870-017-0990-x (PMC5329940; doi:10.1186/s12870-017-0990-x)
Supplement: Additional file 5 Figure S3. — Characteristics of the homology search of Taihangia unigenes against the NR database. (PDF 136 kb) [file 12870_2017_990_MOESM5_ESM.pdf]

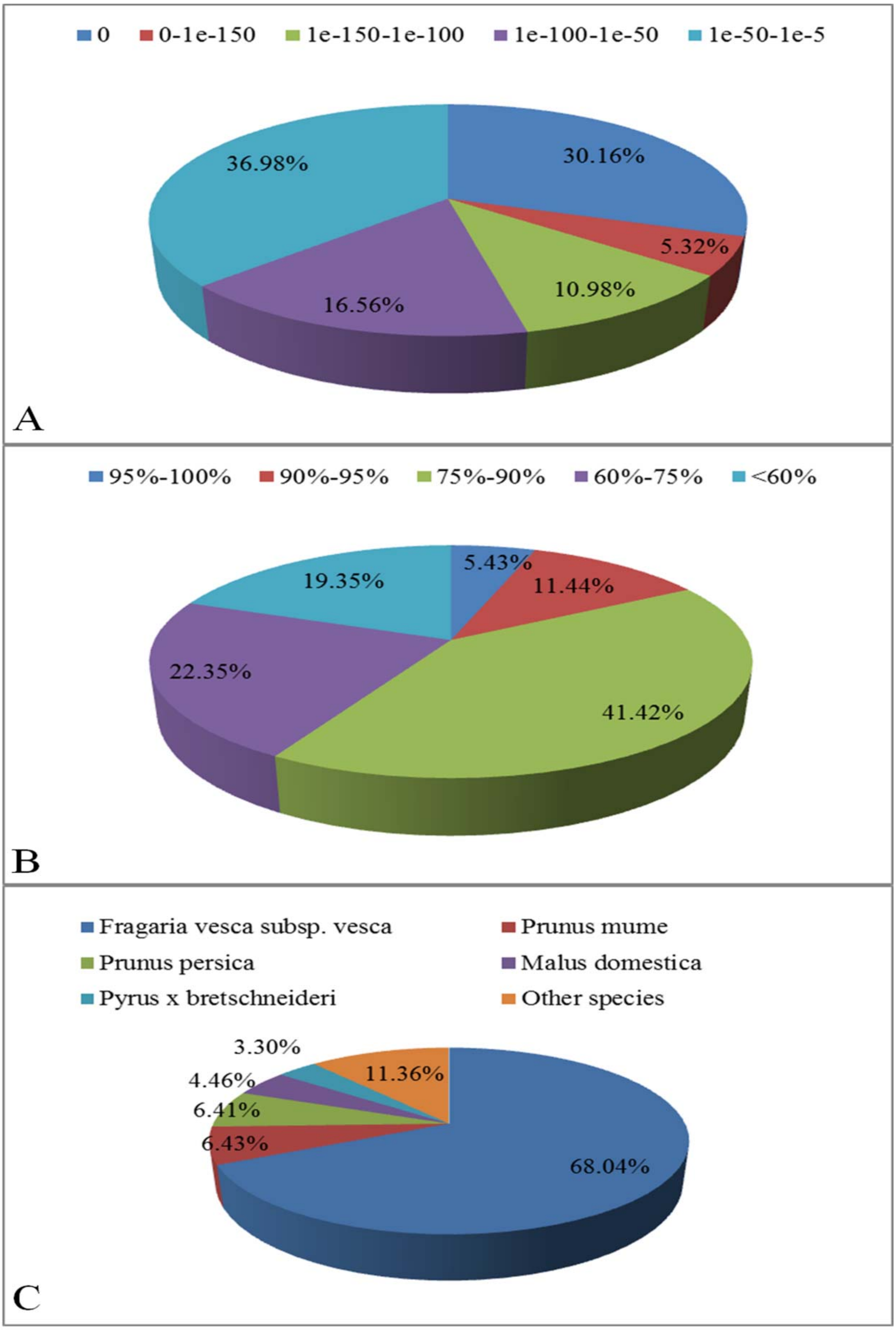

Figure S3. Characteristics of homology search of Taihangia unigenes against the NR database. The cufoff value was set at 1e-5. (A) e value distribution of the top Blast hits for each unigene; (B) Similarity of the best Blast hits for each unigene; (C) Species distribution of the top Blast hits.
