# Supplementary material for: De novo sequencing and comparative transcriptome analysis of the male and hermaphroditic flowers provide insights into the regulation of flower formation in andromonoecious taihangia rupestris
Source: BMC Plant Biol. 2017 Feb 28;17:54. doi: 10.1186/s12870-017-0990-x (PMC5329940; doi:10.1186/s12870-017-0990-x)
Supplement: Additional file 8: Table S3. — Taihangia MADS-box gene names and attributes. (DOCX 17 kb) [file 12870_2017_990_MOESM8_ESM.docx]

Table S3 *Taihangia rupestris* MADS-box gene names and attributes.

| Gene Name | Transcriptome ID | Additional name | GenBank accession | Strand | Protein length | Subfamily |
| --- | --- | --- | --- | --- | --- | --- |
| TruMADS1 | c22225.graph_c0 | TrSHP | DQ248949 | + | 257 | AG |
| TruMADS2 | c26730.graph_c0 | - | - | + | 75 | AGL12 |
| TruMADS3 | c26895.graph_c0 | TrSEP1 | DQ372969 | + | 252 | SEP |
| TruMADS4 | c28960.graph_c0 | TrAG | DQ248948 | - | 198 | AG |
| TruMADS5 | c30292.graph_c0 | TrPI | DQ248947 | - | 210 | PI/AP3 |
| TruMADS6 | c31824.graph_c0 | TrTM6 | DQ248946 | - | 239 | PI/AP3 |
| TruMADS7 | c32275.graph_c0 | - | - | - | 239 | SOC |
| TruMADS8 | c32480.graph_c0 | - | - | - | 87 | Bs |
| TruMADS9 | c33142.graph_c0 | - | - | - | 130 | MIKC^*^ |
| TruMADS10 | c34320.graph_c0 | - | - | - | 212 | SOC |
| TruMADS11 | c39130.graph_c0 | - | - | + | 276 | SVP/AGL24 |
| TruMADS12 | c46770.graph_c0 | - | - | + | 139 | MIKC^*^ |
| TruMADS13 | c14107.graph_c0 | - | - | - | 218 | AP1/FUL |
| TruMADS14 | c14190.graph_c0 | - | - | - | 117 | SOC |
| TruMADS15 | c14751.graph_c0 | - | - | + | 198 | SVP/AGL24 |
| TruMADS16 | c15527.graph_c0 | TrMADS3 | EF469601 | + | 208 | FLC |
| TruMADS17 | c8824.graph_c0 | - | - | + | 247 | AGL6 |
| TruMADS18 | c9146.graph_c0 | - | - | + | 282 | AP1/FUL |
| TruMADS19 | c16469.graph_c0 | - | - | - | 377 | MIKC^*^ |
| TruMADS20 | c16489.graph_c0 | - | - | + | 250 | AG |
| TruMADS21 | c18217.graph_c0 | - | - | + | 86 | AP3/PI |
| TruMADS22 | c20209.graph_c0 | - | - | - | 208 | MIKC^*^ |
| TruMADS23 | c20350.graph_c0 | TrSEP3 | DQ372971 | + | 249 | SEP |
| TruMADS24 | c16920.graph_c0 | TrFUL | DQ248945 | - | 150 | AP1/FUL |
| TruMADS25 | c37570.graph_c0 | - | - | + | 530 | MIKC^*^ |
