# Supplementary material for: De novo sequencing and comparative transcriptome analysis of the male and hermaphroditic flowers provide insights into the regulation of flower formation in andromonoecious taihangia rupestris
Source: BMC Plant Biol. 2017 Feb 28;17:54. doi: 10.1186/s12870-017-0990-x (PMC5329940; doi:10.1186/s12870-017-0990-x)
Supplement: Additional file 12: Table S4. — The selected DEGs, IDs, and primer pairs for RT-qPCR. (DOCX 18 kb) [file 12870_2017_990_MOESM12_ESM.docx]

Table S4. The selected DEGs, IDs, and primer pairs for RT-qPCR.

| Code | Primer sequence (5’ – 3’) | Product size (bp) | Transcriptome ID | Gene |
| --- | --- | --- | --- | --- |
| Tru01 | F: GACGAAAACAGGCGATAAAG | 265 | c12432.graph_c0 | uncharacterized protein |
|  | R: GTAAAGGAAGAAAGCAAGGAAT |  |  |  |
| Tru02 | F: AATGGAGGCAGCAACAGA | 80 | c13287.graph_c0 | caffeic acid O-methyltransferase |
|  | R: CTACAAAGCCCAGAATGAAC |  |  |  |
| Tru03 | F: TTGGCGGCTTCATTTCTC | 159 | c15544.graph_c0 | tryptophan synthase alpha chain-like |
|  | R: TCAACTTTCTTGCCCTATCACT |  |  |  |
| Tru04 | F: CATTACGCAATCAATCCTCCCT | 251 | c16428.graph_c0 | defensin-like protein 2-like |
|  | R: CCACCATCCCAGTAGCCAAG |  |  |  |
| Tru05 | F: GTGGTGCGTTTGTAATCTCG | 141 | c16472.graph_c0 | cytochrome P450 82A3-like |
|  | R: TTCTGTTTTGCTTATGCTCGT |  |  |  |
| Tru06 | F: GGTGAGGCAGGCAAGAAAAT | 145 | c16542.graph_c0 | pectinesterase/pectinesterase inhibitor 28 |
|  | R: GTGATGAGGCGAGGAGGC |  |  |  |
| Tru07 | F: ACTGTGCGATTGGAAATAACTG | 123 | c17750.graph_c0 | transcription factor bHLH135-like |
|  | R: GGTGGATGACCTAAGCGAGA |  |  |  |
| Tru08 | F: CCAAAGCACAGGTTTACAAG | 171 | c25059.graph_c0 | F-box/kelch-repeat protein |
|  | R: AGGAGACCACCAACAATAGAC |  |  |  |
| Tru09 | F: CACAATAGTCTTCGCACCT | 136 | c29511.graph_c0 | peroxidase 3-like |
|  | R: AACAACGACAGTAGAAATGG |  |  |  |
| Tru10 | F: GCTGATTAGGCTGGATAGAA | 235 | c32480.graph_c0 | MADS-box protein FBP24-like |
|  | R: AAACAGAAGGAGGACGAGA |  |  |  |
| Tru11 | F: TAATCCAGGAGCCCCACT | 249 | c32665.graph_c0 | Dof zinc finger protein DOF5.6-like |
|  | R: TTTTCGCATCTCAATCACTT |  |  |  |
| Tru12 | F: ATGGAAAACCAAAACGAAGG | 101 | c32844.graph_c0 | receptor-like protein kinase ANXUR2-like |
|  | R: AGTAGACCAGGCAAGAGCAAG |  |  |  |
| Tru13 | F: GCGGAGACGGTAGAATAGTTA | 128 | c35962.graph_c0 | sucrose transport protein SUC2-like |
|  | R: GTTTGTATCGGTGGTGAGTG |  |  |  |
| Tru14 | F: AACGGTTTCTGCCTCTGTGC | 158 | c8145.graph_c1 | growth-regulating factor 5-like |
|  | R: TTTTGGGTGGGGATGCTATG |  |  |  |
| Tru15 | F: GCCAAAGGATACGCTCAAGA | 187 | c8873.graph_c0 | putative RING-H2 finger protein ATL21A-like |
|  | R: ATCACAACCGACCACAGAAAC |  |  |  |
| Tru16 | F: AATCCGAATCCCAGAAAACC  R: GGAAGAACAGCCTACCCACC | 124 | c9479.graph_c0 | ribonuclease |
| EF1α | F: CCCTGGGCAGATTGGAAACG  R: CCTCACAGCAAAGCGACCGA | 235 | c16607.graph_c0 | elongation factor 1 alpha |
| UBQ | F: GTCTACAAGGACCACATCACT  R: CTTCTTATTTACGACACCGA | 135 | c38440.graph_c0 | polyubiquitin |
